# Large-scale analysis of MicroRNA expression in motor neuron-like cells derived from human umbilical cord blood mesenchymal stem cells

**DOI:** 10.1038/s41598-022-09368-6

**Published:** 2022-04-07

**Authors:** Davood Sanooghi, Abolfazl Lotfi, Zohreh Bagher, Shirin Barati, Afzal Karimi, Faezeh Faghihi, Erfan Lotfi, Mohammad Taghi Joghataei

**Affiliations:** 1 Cancer Research Center, Shahid Beheshti University of Medical Sciences, Tehran, Iran; 2 Agricultural College of Damavand, Technical and Vocational University, Tehran, Iran; 3 ENT and Head & Neck Research Center and Department, The Five Senses Institute, Hazrat Rasoul Akram Hospital, Iran University of Medical Sciences, Tehran, Iran; 4 Department of Anatomy, Saveh University of Medical Sciences, Saveh, Iran; 5 Department of Medical Biotechnology, School of Advanced Technologies in Medicine, Iran University of Medical Sciences, Tehran, Iran; 6 Cellular and Molecular Research Center, Iran University of Medical Sciences, Tehran, Iran; 7 Pad Nahad Tabiat Company, Ltd., Tehran, Iran; 8 School of Medicine, Birjand University of Medical Sciences, Birjand, Iran; 9 Department of Neuroscience, Faculty of Advanced Technologies in Medicine, Iran University of Medical Sciences, Tehran, Iran

**Keywords:** Molecular biology, Neuroscience, Stem cells

## Abstract

Motor neuron diseases such as spinal cord injuries and amyotrophic lateral sclerosis are known as the most common disorders worldwide. Using stem cells (e.g., human umbilical cord blood mesenchymal stem cells) is currently a potent medical approach for modulating the impact of neural damages and regeneration of spinal cord injuries. MicroRNAs (miRNA) are taken into account as principal regulators during differentiation. The miRNAs play a significant role in stem cell self-renewal and fate determination. There are few studies on how miRNAs regulate neural differentiation in stem cells. The purpose of this study is to explore miRNA profiles of CB-MSCs during differentiation into motor neuron-like cells. Human CB-MSCs were isolated and characterized using flow cytometry. Cell differentiation has been induced by combining retinoic acid (RA) and sonic hedgehog (Shh) in a two-step protocol for 14 days. Then, cell differentiation was confirmed by immunocytochemistry and flow cytometry. The miRNA was analyzed using Illumina/Solexa sequencing platform. In this regard, three libraries were prepared to investigate the effect of these two biological morphogens on the miRNA profile of the differentiating cells. These libraries were Control (non-treated CB-MSCs), Test 1 (RA + /Shh +), and Test 2 (RA-/Shh-). Quantitative RT-PCR was employed to verify miRNA expression. CB-MSCs were spindle-shaped in morphology, and they did not express hematopoietic markers. After differentiation, the cells expressed motor neuron markers (i.e., Islet-1, SMI-32, and ChAT) at the protein level after 14 days. The analysis of miRNA sequencing demonstrated a significant up-regulation of miR-9-5p and miR-324-5p in Test 1 (RA + /Shh +). Also, there is a considerable down-regulation of mir-137 and let-7b in Test 2 (RA-/Shh-). These results have been obtained by comparing them with the Control library. Indeed, they were responsible for neuron and motor neuron differentiation and suppression of proliferation in neural progenitor cells. Furthermore, significant up-regulation was detected in some novel microRNAs involved in cholinergic, JAK-STAT, and Hedgehog and MAPK signaling pathways. CB-MSCs are potent to express motor neuron markers. This procedure has been performed by developing a two-week protocol and employing Shh and RA. The miRNA profile analysis showed a significant up-regulation in the expression of some miRs involved in neuron differentiation and motor neuron maturation. MiR-9-5p and miR-324-5p were up-regulated at the early stage of differentiation. Also, miR-137 and miR-let-7b were downregulated in the absence of RA and Shh. Furthermore, several novel miRNAs involved in cholinergic, Hedgehog, MAPK, and JAK-STAT signaling pathways have been detected. However, further studies are still necessary to validate their functions during motor neuron generation and maturation.

## Introduction

The miRNAs represent a group of non-coding RNAs that consist of 18–24 nucleotides regulating post-transcriptional gene expression^1^. These regulators play a principal role in stem cell self-renewal and fate determination. In this regard, crucial tasks are defined for gene regulation. This procedure is performed by degrading messenger RNAs or inhibiting gene translation^2^.

Every miRNA has the potential to identify multiple target mRNAs^3^. Thus, the expression of a series of genes that control biological processes is modulated by one miRNA. It plays a crucial role in cellular processes such as differentiation and cell proliferation during normal development^4^. It is due to its capacity for coordinating the control of gene expression and the broad range of expression across different types of cells.

In recent years, different investigations have attempted to identify the link between miRNAs and neurogenesis. The results demonstrated that non-coding RNAs are crucial components to modulate transcriptional networks associated with neural development and function^5^. For example, miR-9 transiently expression during motor neuron differentiation regulates the expression of FoxP1, and a HOX accessory factor coordinates motor neuron subtype identity and connectivity. In mice, miR-9 overexpression induces neuronal differentiation by inhibiting the nuclear TLX receptor^15^. A positive regulator of the Wnt signaling pathway^16^ is essential for neuronal progenitor self-renewal. Also, let-7b and miR-137 are principal regulators for neural stem cell proliferation. These are significantly up-regulated during neural differentiation. Also, they suppress the expression of TLX^22,23^.

There are few protocols to induce stem cell-derived motor neurons. For example, transplantation of BDNF-overexpressing hUC-MSC-derived motor neurons could improve motor performance and prolong the survival of the amyotrophic lateral sclerosis (ALS) model in mice. In the developing neural tube, the generation of motor neurons along with the rostrocaudal axis is spatiotemporally orchestrated by a set of morphogens such as sonic hedgehog (Shh) and retinoic acid (RA)^6^. RA and Shh play a principal role in specifying motor neurons in the central nervous system^7^. According to in-vitro studies, the administrations of RA^8^ and Shh^9^ support the differentiation of Wharton's jelly, olfactory mucosa^10^, bone marrow, and chorion-derived mesenchymal stem cells into motor neuron-like cells^11^. It appears that RA and Shh regulate the expression of transcription factors, which are essential for specifying motor neurons in the spinal cord and a synergistic manner^11^.

The downstream molecular interactions of RA and Shh signaling pathways are often unclear. However, it seems that post-transcriptional silencing carried out by miRNAs has a vital role in the spatiotemporal regulation of neuronal specification^12^. The study conducted by Hohjoh and Fukushima regarding the up-regulation of 19 miRNAs during neurogenesis in mouse and human cells after RA treatment indicated that their functions are conserved in mammalian species^13^. For example, they reported that miR-302^14^ and miR-124a exhibited the opposite expression patterns in response to RA treatment at the beginning of neural differentiation. Moreover, the suppression of some miRNAs (e.g., miR-17-3p by Shh) directs the differentiation of neuronal progenitors into motor neurons and decreases the interneuron population^15^.

Many types of researches have been performed in the field of neurogenesis. However, there is little knowledge related to the role of miRNAs in the neurogenesis process. Indeed, these researches have failed to portray a dynamic profile of the miRNA expression at the time of neurogenesis^4^. Despite the concerns of neuroscientists about the application of stem cells in neurogenesis, few studies have been performed on how miRNAs regulate neural differentiation in stem cells. Therefore, studies on miRNA profiles of differentiating stem cells are beneficial to identify the effect of potent miRNAs on neurogenesis.

Human umbilical cord blood (UCB) is a postpartum medical waste product. It is known as a rich source of stem cells. The UCB has several advantages over other sources of stem cells in regenerative medicine, including convenient access, a higher amount of pluripotent cells, higher toleration across the barrier, and a slighter prevalence of graft-versus-host disease (GVHD)^16^. The hUCB-MSCs are known as proper cell candidates for regenerating the lost cells in the central nervous system^17^. These cells have self-renewal features^18^, immunomodulatory properties, and neural differentiation capacities. This study is focused on differentiating human CB-MSCs into motor neuron-like cells and examining the miRNA profile of the motor neuron-like cells derived in the presence of RA and Shh. In this study, the surface markers of CD90, CD44, and CD73 expressed by hMSC and specific markers (i.e., CD34 and CD45 expressed by hematopoietic stem cells) were characterized as the negative markers for the MSCs^19^. In this study, the differentiation was induced by RA and Shh and applying the proposed two-step protocol. In this regard, the results revealed that the cells could express motor neuron markers (i.e., Islet-1, SMI-32, and ChAT) at the protein level. The miRNA sequencing showed a significant up-regulation in the expression of miR-9-5p and miR-324-5p at the early stage of the differentiation. This procedure occurred in the presence of RA and Shh (RA + /Shh + test group). The miR-137 and let-7b were down-regulated in Test 2 (RA-/Shh-). It was confirmed by comparing the achieved results with the Control library. They were all responsible for neuron and motor neuron differentiation and suppression of proliferation in neural progenitor cells, respectively. Also, we could detect some novel miRNAs involved in cholinergic, JAk-STAT, and Hedgehog and MAPK signaling pathways during differentiation. Further experiments are still essential to validate their expression and functions during motor neuron generation and maturation.

## Materials and methods

### Cell culture

After gaining informed consent from mothers, we gathered hUCB-MSCs from the umbilical vein of infants delivered by elective cesarean. Using cells for this study was endorsed by the Ethical Committee of Iran University of Medical Sciences. The hUCB-MSCs were isolated based on pre-defined protocols^20^. In this study, we collected blood samples using a 50 ml sterile syringe that contained 10 µl of heparin. Then, the samples were moved to the lab and placed on an ice pack. Almost four hours after collecting samples, red blood cells have been removed by adding 1 ml of 10% Hydroxyethyl starch (Fresenius, Germany). Then, the supernatant was collected and washed with phosphate-buffered saline (PBS, 5 ml). Next, it was supplemented with 100 U/ml penicillin, 0.2% EDTA, 15% fetal bovine serum (FBS) (Gibco, Germany), and 0.1 mg/ml streptomycin (Sigma, USA). After centrifugation over Ficoll-Hypaque (the ratio of 1:1, Germany) at a rate of 300 g for 15 min, mononuclear cells were achieved and cleansed in PBS. Afterward, a total of 5 × 10^3^ cells/cm^2^ were cultured in 75 cm^2^ tissue culture flasks. These flasks contained DMEM-F12 with 100 μg/ml streptomycin, 10% FBS, and 100 U/ml penicillin (all purchased from Gibco, Germany). These were placed in a dampened chamber at 37 °C with 5% CO_2_. The medium was refreshed one week later. After observing the first colonies, the medium was changed every day. Afterward, at 60% confluence, new culture flasks were used to plate cells, and the medium was modulated every three days. The following experiments contained cells at passage three.

### Differentiation into motor neuron-like cells

The hUCB-MSCs were induced into motor neuron-like cells based on the protocols defined in the previous step ^20^. A total of 1 × 10^5^ cells were seeded in every well from a 24-well culture plate and incubated at dampened chamber overnight. On the next day, the expansion medium was replaced by the medium used before induction. This medium contained 20% fetal bovine serum, DMEM-F12, 10 ng/ml of fibroblast growth factor (Sigma, USA), 250 mM of isobutylmethylxanthine (IBMX), 2% B27 (Gibco, Germany), and 100 mM of β-mercaptoethanol. We retained the cells in a dampened chamber overnight. On the next day, they were treated with the induction medium that contained DMEM-F12 with 0.01 mM of all-trans RA (Sigma, USA), 100 ng/ml supplementation of Shh (R&D, USA), and 0.2% B27 (Invitrogen, USA) for a week (RA + /Shh + , known as Test 1 group). Then, the RA and Shh were removed at the end of the first week. Afterward, the medium was substituted with DMEM-F12 and supplemented with 100 ng/ml of brain-derived neurotrophic factor (BDNF) (Invitrogen, USA) and 0.2% B27 as surviving factor for one more week (RA-/Shh-, known as Test 2 group). A summary of the protocol is illustrated in flow chart.
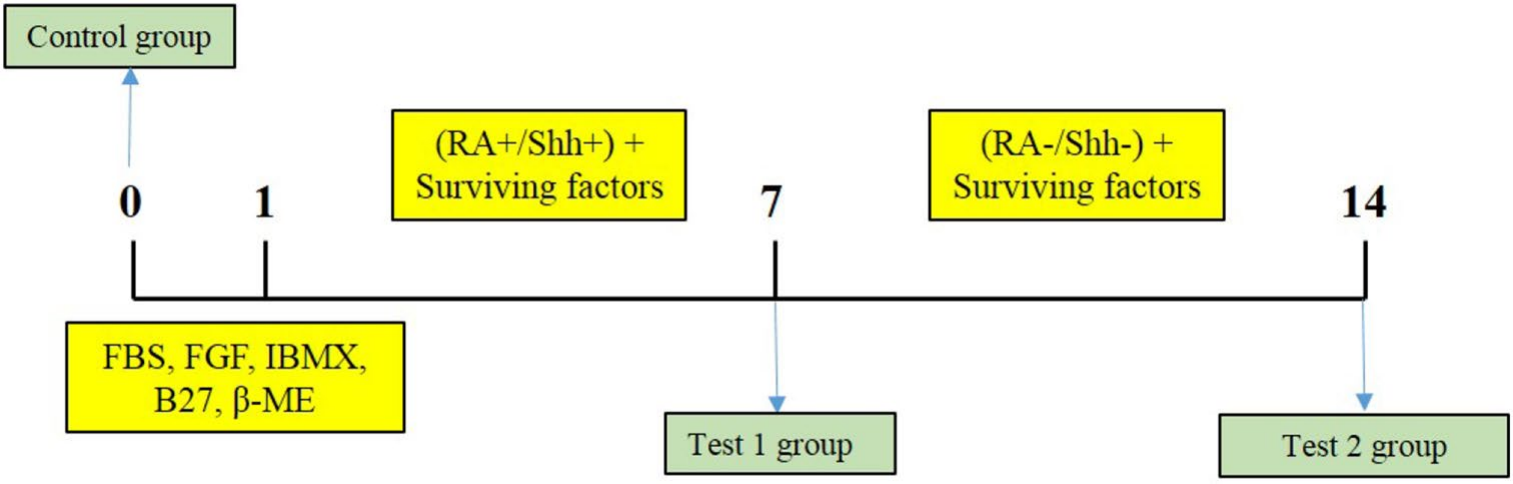


This flow chart is showing the study design and the experimental groups. Test 1 is the group of MSCs treated with RA/Shh collected for analysis at day 7. Test 2 is the same group of MSCs which was kept in the culture medium without RA/Shh for addition of 7 days. Fetal bovine serum (FBS), fibroblast growth factor (FGF), isobutylmethylxanthine (IBMX), surviving factors (B27 + BDNF) and β-mercaptoethanol (β-ME).

### Immunostaining: flow cytometry and immunocytochemistry

#### Characterization of hUCB-MSCs

Mesenchymal stem cells were characterized by mono-color cytofluorimetric analysis. This process was carried out based on the protocol defined by the authors in the previous study^20^. In this regard, we incubated 1 × 105 cells with 10% goat serum in PBS at a temperature of 4 °C. After spending one hour, the serum was withdrawn, and the incubation of cells was performed either by phycoerythrin (PE)-conjugated monoclonal antibodies or fluorescein isothiocyanate (FITC) against human CD34, CD45, CD44, CD73, and CD90 (all of them were from BD bioscience; cat#348,057, cat#347,463, cat#347,943, cat#561,014, cat#561,970) at 4 °C for 40 min. All antibodies were diluted in PBS/0.1%BSA at the concentration of 1:200. Also, the control library was comprised of isotype-matched antibodies. The expression of antigens was detected using BD FACSCalibur. In this study, the data analysis was performed by FlowJo v7.6.1 software.

#### The expression of motor neuron-related markers

Cytofluorimetric analysis has quantitatively been carried out to identify the expression of Islet-1, ChAT, and SMI-32 antigens at the end of the second week (T2 time point). At the end of the proposed two-week protocol, the differentiated cells were fixed in 4% paraformaldehyde for 4 h. The permeabilization analysis was performed by incubating the cells with 0.2% Triton X-100 in PBS (Gibco, Germany) at room temperature. The cells were washed by PBS twice, and then they were incubated with 10% goat serum in PBS for 60 min at room temperature. Afterward, samples were incubated with primary antibodies (1:200) against human ChAT (Abcam, USA, cat#34,419), SMI-32 (Abcam, USA, cat# 5.59844.0001), and Islet-1 (Santacruz, USA, cat#530,265) at 4 °C. In the next step, the cells were washed by PBS twice. Then, we conducted incubation using the matching phycoerythrin (PE) or fluorescein isothiocyanate (FITC) conjugated secondary antibodies (Sigma, USA; 1:100) for 45 min at 37 °C. The expression of antigens was detected by BD FACSCalibur (BD Biosciences) at the end of the defined two weeks protocol. In this context, positive expression was defined as a fluorescence level above 95% compared to the one measured by the corresponding isotype-matched control antibody. Data were analyzed by Flowjo v7.6.1 software. For quantification of the flow cytometry data, the expression level of each three markers; including ChAT, Islet-1, and SMI-32 was investigated in three different biological replicates (*n* = 3) and the average of the expression level of each marker was quantified as mean ± standard error of mean. Also, the expressions of Islet-1, ChAT, and SMI-32 proteins were observed by Olympus DP70 fluorescent microscope after nuclei staining with DAPI (Sigma, USA). It was performed based on the mentioned immunostaining protocol^10^.

### The expression of motor neuron-related genes

Real- time- PCR was carried out to evaluate the expression of motor neuron- related genes at T1 and T2. Similar to our previously set up protocol (PMID: 28,153,469), total cellular RNA was extracted using TRIzol reagent (Invitrogen, Germany). After digestion of DNA, the purity of RNA was quantified. Complementary DNA (cDNA) was synthesized using RevertAid H Minus First Strand cDNA Synthesis Kit (Fermentase, Canada). Real-time PCR reactions were carried out using 7500 Real time PCR system (Applied Biosystems, USA). In each PCR reaction, X1 SYBR Green PCR Master Mix (Applied Biosystems, USA) was mixed with 12 ng of cDNA and the related primers (supplementary data 1a) in a total volume of 20 μl. The expression of GAPDH, as an internal control was used to normalize the expression levels of the target genes in the treated cell groups in comparison with the same genes in human CB-MSCs at passage three.

### MicroRNA sample preparation for sequencing

In this experiment, three libraries have been provided, including “Control”, “RA/Shh^+^”, and “RA/Shh^–^”. In this case, a total of 1 × 10^5^ cells were seeded in each 24-well culture plate and incubated at dampened chamber overnight. On the next day, the expansion medium was replaced by the medium used before induction. This medium contained 20% fetal bovine serum, DMEM-F12, 10 ng/ml of fibroblast growth factor (Sigma, USA), 250 mM of isobutylmethylxanthine, 2% B27 (Gibco, Germany), and 100 mM of β-mercaptoethanol. The cells have been retained in a dampened chamber overnight. On the next day, these were treated with the induction medium for a week. It contained DMEM-F12 with 100 ng/ml supplementation of Shh (R&D, USA), 0.01 mM of all-trans RA (Sigma, USA), and 0.2% B27 (Invitrogen, USA). The cells were collected and named as “RA^+^/Shh^+^ group” or Test 1. Afterward, the remaining cells have been kept in the medium for one more week. The medium was contained DMEM-F12 without RA and Shh and supplemented with survival factors, including 100 ng/ml of BDNF (Invitrogen, USA) and 0.2% B27. The cells were collected and named as “RA^–^/Shh^–^ group” or Test 2. For sequencing, the sample size was ≥ 1 µg, the concentration was ≥ 50 ng/µl, and the sample volume was about 15 µl. These quantities were considered based on BGI's general sample requirement guideline. Similar quantities of total RNA from labeled control and two test groups (RA/Shh^+^ and RA^–^/Shh^–^) were pooled, and the sample has been run in the sequencer.

#### RNA extraction

The RNA extraction kit has been used to extract total RNA from the cell groups (SV Total RNA Isolation System, USA). This procedure was accomplished based on the instructions provided by the manufacturer. After incubation, genomic DNA was withdrawn using RNase-Free DNase (TaKaRa, Otsu, Japan). This process was followed by an RNA Clean Purification Kit (BioTeke, Beijing, China). This procedure was performed for 15 min at a temperature of 37 °C. Also, Agilent 2100 Bioanalyzer was utilized to assess the quality and integrity of RNAs. The purification of total RNA has been performed using the electrophoretic separation on a 15% denaturing polyacrylamide gel. This process was followed by excision and recovery of small RNA regions (15–30 nucleotide bands). Then, the sequences of proprietary adapters were ligated to the 5′- and 3′-termini of these small RNAs. Similar quantities of total RNA from labeled control and two test groups (RA/Shh^+^ and RA^–^/Shh^–^) were pooled. The purified gel products of the ligation have been converted into DNA. Then, the outcome was amplified by RT-PCR with 15 PCR cycles to create libraries. They were sequenced by Illumina/Solexa platform sequencer at Beijing Genomics Institute (BGI), Hong Kong.

### Prediction of conserved and novel miRNAs

Extensive raw sequences are collected from high-throughput sequencing. Following the removal of impure sequences (adaptor reads, low-quality reads, and reads that were either less than 18 or greater than 30 in length), we queried unique reads against non-coding RNAs such as rRNAs, tRNAs, small nucleolar RNAs (snoRNAs), and small nuclear RNAs (snRNAs) in the Rfam (http://www.sanger.ac.uk/Software/Rfam) and NCBI GenBank (http://www.ncbi.nih.gov/GenBank/) databases. The miRNA database (miRBase 20.0) has been utilized to detect preserved miRNAs. In this case, a maximum of two mismatches was allowed. The Mireap software (https://sourceforge.net/projects/mireap/) has been employed to detect novel miRNAs. Thus, the novel miRNAs were predicted by the rest of the unknown small RNAs (sRNAs). The principal criteria are adopted to screen the potential novel miRNAs. In this study, Mfold was utilized (http://mfold.rna.albany.edu/?q=mfold/RNA-Folding-Form) to construct the stem-loop structures for putative pre-miRNAs. If a complete stem-loop structure was formed, the sRNA sequence would have landed at a stem arm together with other ensuing criteria. Thus, this sRNA was taken into account as a new miRNA.

### MicroRNA target genes prediction and functional annotation

In this section, the miRanda program has been considered to predict target genes for the identified miRNAs. The candidate targets were BLASTn hits that had less than four mismatches. Also, the NCBI database and target sequences were utilized to predict the functions of the candidate targets by BLASTX. The enrichment analysis of gene ontology (GO) (http://www.geneontology.org/) was employed for functional annotation in the candidate target gene of miRNAs. The Kyoto Encyclopedia of Gene and Genome (KEGG) pathway database (http://www.kegg.jp/kegg/) has been employed to detect remarkably enriched signal transduction or metabolic pathways in target gene candidates and compare them with the whole reference gene background. The pathway analysis shed further light on the biological functions of target genes.

### Pattern of expression and cluster analysis

We analyzed the reads of conserved and novel miRNAs derived from the three libraries. This procedure has been implemented to understand the responsive miRNAs in Test 1 and Test 2 groups. The normalized read count has been computed using the following formula:

$${\text{Normalized}}\;{\text{read}}\;{\text{count}} = \left( {{\text{actual}}\;{\text{miRNA}}\;{\text{count}}\;{\text{of}}\;{\text{reads}}/{\text{total}}\;{\text{count}}\;{\text{of}}\;{\text{clean}}\;{\text{reads}}} \right) \times 1000000$$

Also, the expression fold-change was calculated by the following formula:

$${\text{Fold}}\;{\text{change}} = \log 2({\text{A}}/{\text{B}})$$

where A and B represent the normalized read count of a miRNA in A and B libraries.

The *p*-value was computed as follows:

$$p\left( {x|y} \right) = \left( {\frac{{N_{2} }}{{N_{1} }}} \right)\frac{{\left( {x + y} \right)!}}{{x!y!\left( {1 + \frac{{N_{2} }}{{N_{1} }}} \right)^{{\left( {x + y + 1} \right)}} }}$$

$$C\left( {y \le y_{min} |x} \right) = \mathop \sum \limits_{y = 0}^{{y \le y_{min} }} p\left( {y|x} \right)$$

$$D\left( {y \ge y_{max} |x} \right) = \mathop \sum \limits_{{y \ge y_{max} }}^{\alpha } p\left( {y|x} \right)$$

If miRNA has no read in the library, its normalized read count is randomly set to 0.01 for further computations. If the expression level of a miRNA was below 20 reads, the difference analysis is abandoned. The hierarchical cluster analysis was conducted by CLUSTER 3.0 (http://rana.lbl.gov/EisenSoftware.htm) and the TREEVIEW software.

### miRNA verification by QRT- PCR

The real-time PCR was conducted to assess the expression of some novel and conserved miRNAs that were randomly selected. Total RNA was extracted from the three experimental groups (Control, Test 1, and Test 2). This procedure has been carried out using the RNA extraction kit (SV Total RNA Isolation System, USA). Genomic DNA was removed using RNase-Free DNase (TaKaRa, Otsu, Japan), which has been later followed by an RNA Clean Purification Kit (BioTeke, Beijing, China). Also, the reverse transcription and qRT-PCR reactions were performed by DNase-treated RNA samples. A total volume of 20 μL has been considered, and then the reactions contained 5 µM of each primer, 2.5 ng of cDNA, and 10 μL of SYBR Green (Takara, Japan; 1X) considering the cycling profile at 95 °C for 30 s, which was accompanied with 40 cycles at 95 °C and 60 °C for 5 and 20 s, respectively. QRT-PCR was performed in a 7500 real-time PCR system (Applied Biosystems, USA). A triplicate processing of each sample was conducted, and 18SrRNA was adopted as an internal control. The qRT-PCR primers are provided in supplementary 1b.

### Statistical analysis

Data were expressed as the mean ± standard error of mean, and differences between studied groups (*n* = 3 in each group) were statistically assessed by the nonparametric Mann–Whitney test using SPSS ver. 16. *P* values of < 0.05 were considered significant.

### Ethical approval

All experimental procedures were carried out in accordance with the guidelines of the Ethic Committee of Iran University of Medical Science (IUMS), Tehran, Iran.

## Results

### Characterization of hUCB-MSCs

#### Isolation of mesenchymal stem cells from human umbilical cord blood

**Figure 1:**
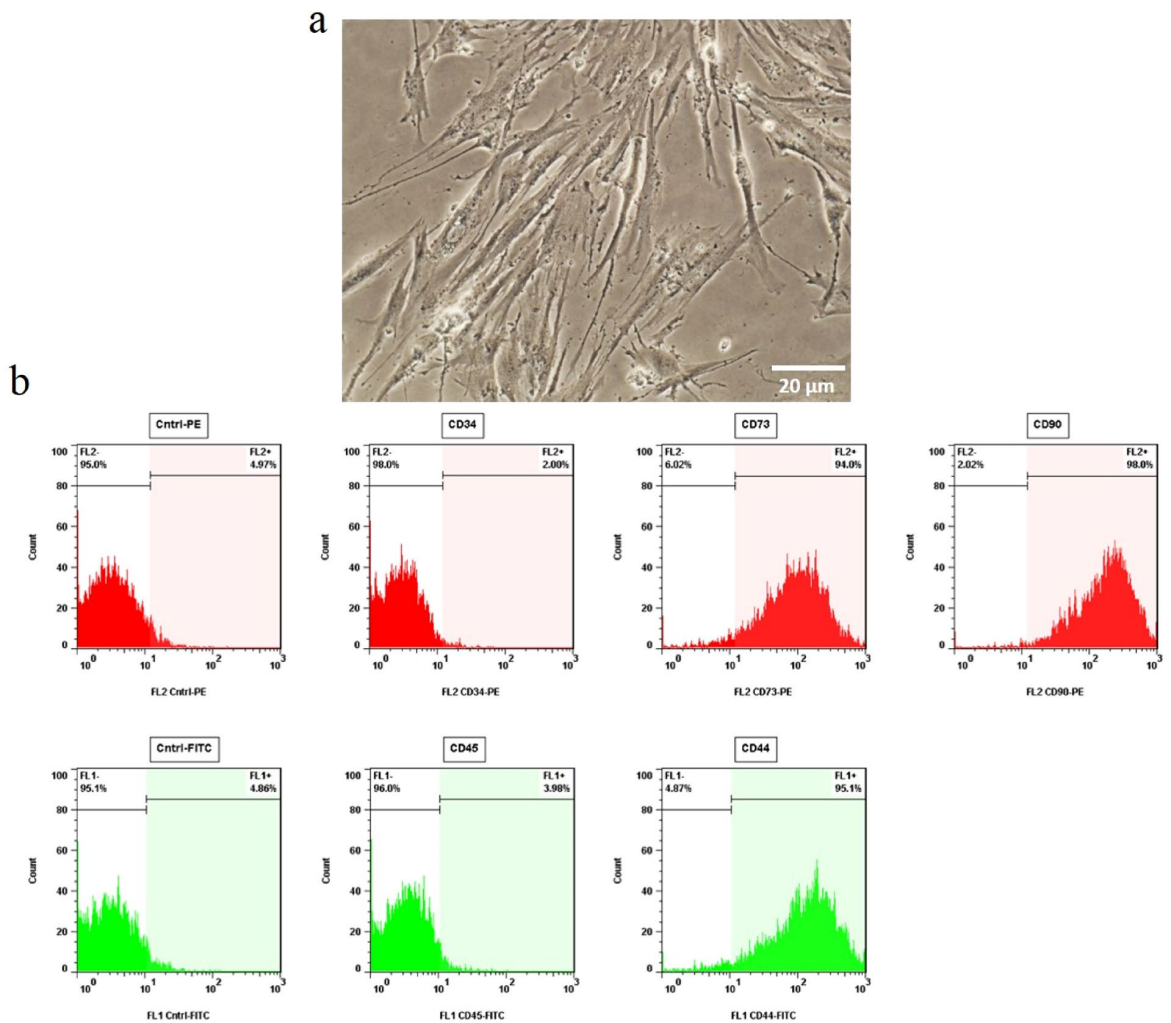
Characterization of human umbilical cord blood mesenchymal stem cells (UCB-MSCs). For characterization of the isolated human UCB-MSCs, cell spindle- shape morphology and the expression of a panel of positive and negative selection markers were investigated. (**a**) Mesenchymal Stem Cells with fibroblast- like morphology are shown at passage three (magnification 20X). (**b**) Representative flow cytometry histograms illustrating the typical expression of CD44, CD73, CD90 antigens by the isolated cells; while a few number of them could express hematopoietic markers such as CD34 and CD45. Positive expression was defined as the level of fluorescence greater than 95% of the one measured using the matched isotype control antibody.

Cell morphology assessment and Flow cytometric analysis have been performed for characterizing the isolated cells. The isolated MSCs taken from human umbilical cord blood had spindle-like morphology at passage three (Fig. 1a).

#### Identification of hUCB-MSCs

The isolated cells were distinguished based on a panel of positive and negative selection markers. They could express CD44, CD73, and CD90 markers on their surfaces at passage three. However, they were negative for hematopoietic antigens such as CD45 and CD34 (Fig. 1b).

### The expression of motor neuron-related markers: flow cytometric analysis and Immunocytochemistry

**Figure 2:**
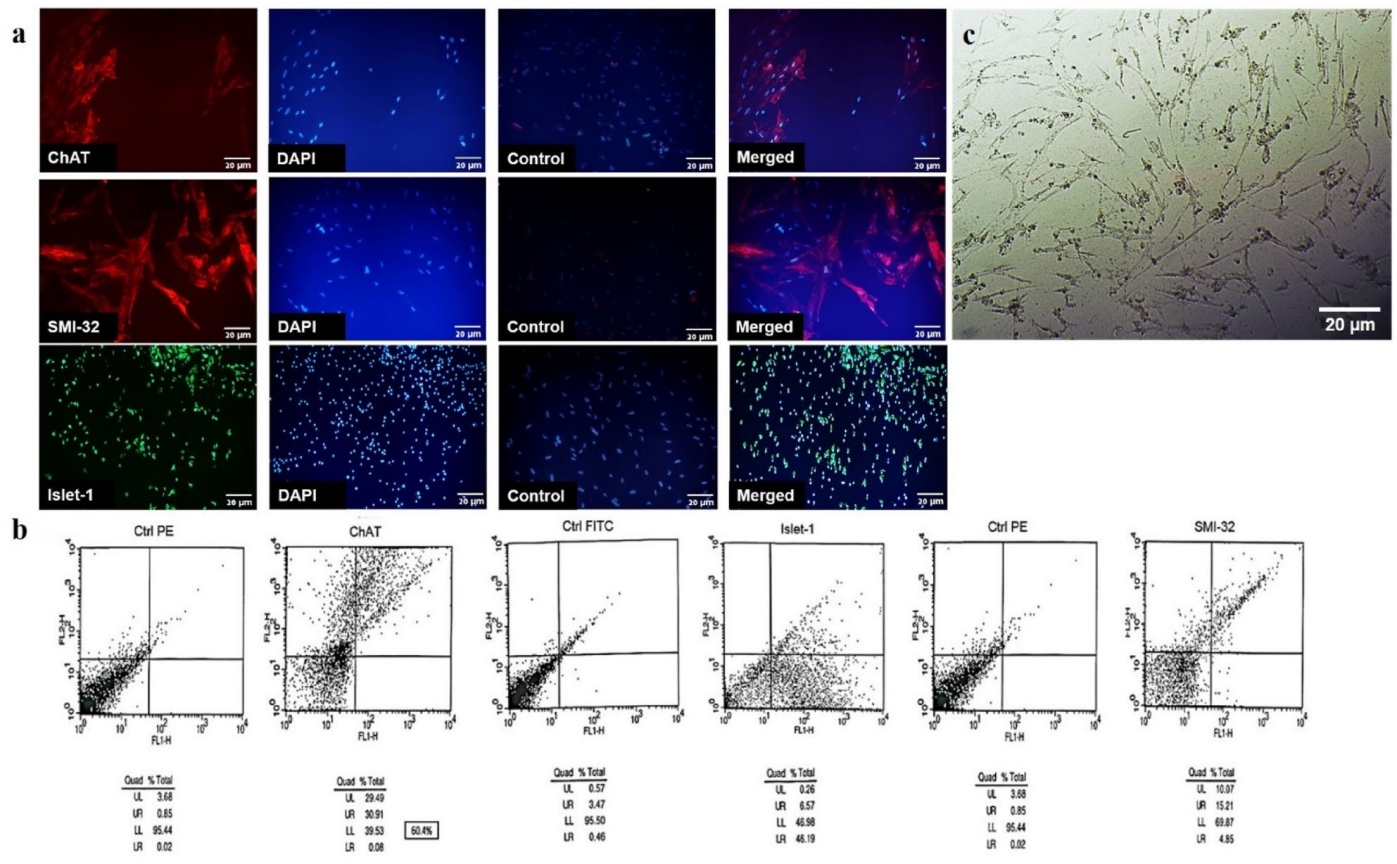
Differentiation of human UCB-MSCs into motor neuron- like cells using RA and Shh. The expressions of motor neuron-related markers were investigated using immunocytochemistry (**a**) and flow cytometry (**b**). The results showed that the cells could express motor neuron- related markers such as ChAT, SMI-32 and Islet-1 after induction with RA and Shh (a: scale bar: 50 µm). (**b**) Representative flow cytometry histograms illustrating the percentage of the cells expressing ChAT, SMI-32 and Islet-1 markers after induction with RA and Shh. Positive expression was defined as the level of fluorescence greater than 95% of the one measured using the matched isotype control antibody. (**c**) Human UCB-MSCs after treatment with our 14 days treatment protocol. A small number of the cells died; while the survived ones displayed the elongated morphology. (scale bar: 50 µm).

The expressions of motor neuron-related markers were investigated using immunostaining two weeks after the beginning of the induction. The results achieved by flow cytometry (Fig. 2a) and immunocytochemistry (Fig. 2b) revealed that after inducing the cells with the defined protocol, they could express Islet-1 (40.427 ± 7.078%), ChAT (47.557 ± 12.475%), and SMI-32 (25.137 ± 3.872%) remarkably at the second week. Quantitative assessment of the expression of the related markers using flow cytometry with three biological repeats (*n* = 3) revealed that 40.427 ± 7.078%, 47.557 ± 12.475%, and 25.137 ± 3.872% of the treated cells could express Islet-1, ChAT and SMI-32 at second week of induction, respectively (Table 1).

**Table 1:** Average of the expression level of each marker.

Marker	Sample 1	Sample 2	Sample 3	Mean	SEM
ChAT	22.61	60.4	59.66	**47.556667**	**12.475162**
SMI-32	25.28	18.36	31.77	**25.136667**	**3.8717969**
Islet-1	28.24	40.28	52.76	**40.426667**	**7.0786942**

Significant values are in [bold].

In this study, we showed human UCB-MSCs morphology after treatment with our 14 days treatment protocol. A small number of the cells died; while the survived ones displayed the elongated morphology (Fig. 2c).

### The expression of motor neuron- related genes

**Figure 3:**
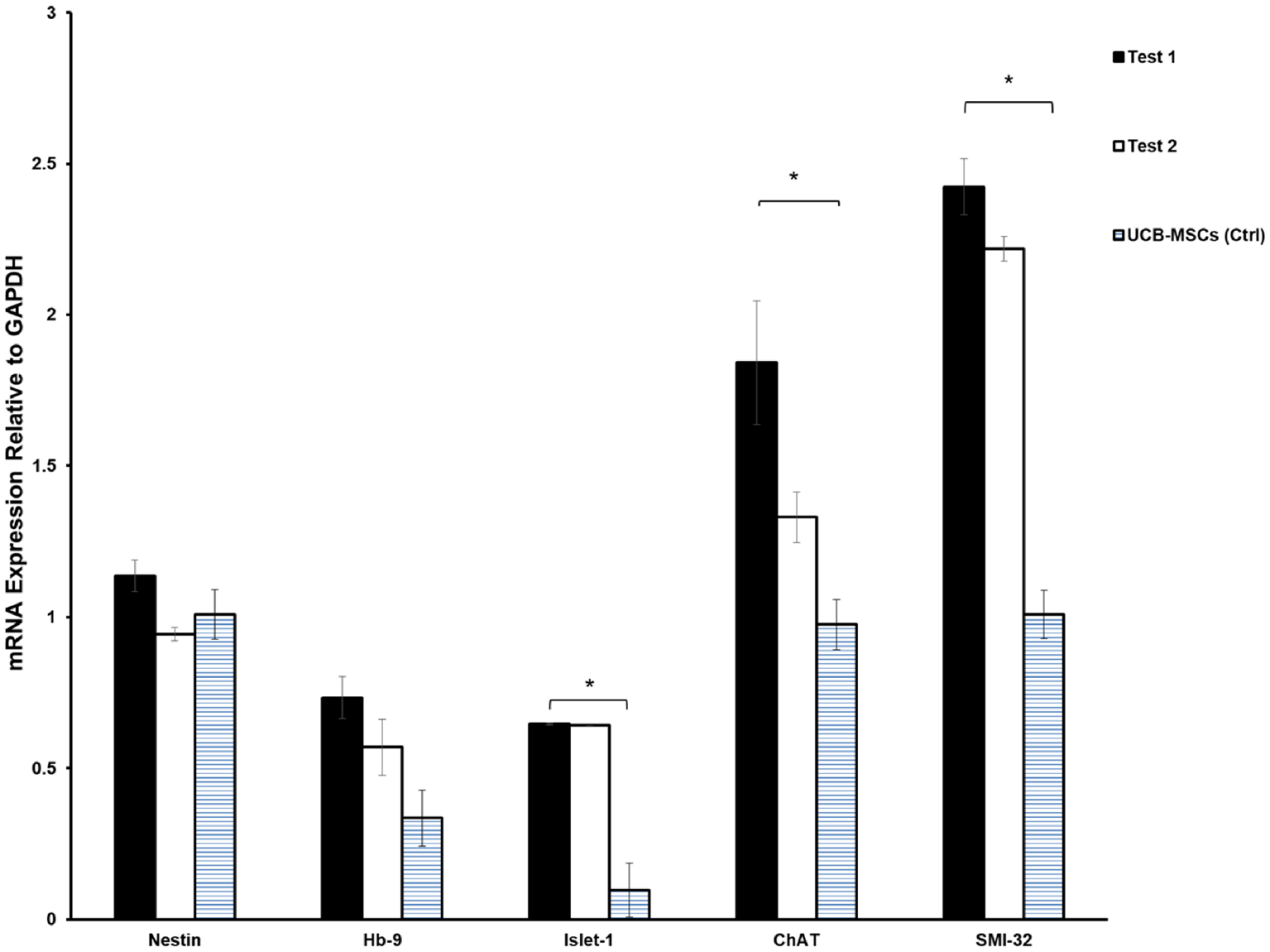
The expression pattern of motor neuron- related markers at the level of mRNA. The significant upregulation of Islet-1, SMI-32 and ChAT was observed in test groups when compared with the control (hUCB-MSCs; *P* < 0.05). No significant upregulation was observed in the expression of HB-9. The expression of Nestin was observed in the treated cells and MSCs as the control group. Values are expressed as the mean ± SEM.

Gene expression analysis revealed that treatment of human CB-MSCs led to the significant upregulation of the genes ChAT, Islet-1 and SMI- 32 in comparison with non- treated human UCB-MSCs (Control) at T1 and T2 (*P* < 0.05). The slight expression of Hb-9 was also detected. No significant difference was observed in the expression of the candid genes between T1 and T2 (Fig. 3).

### Deep sequencing of small RNA libraries

**Figure 4:**
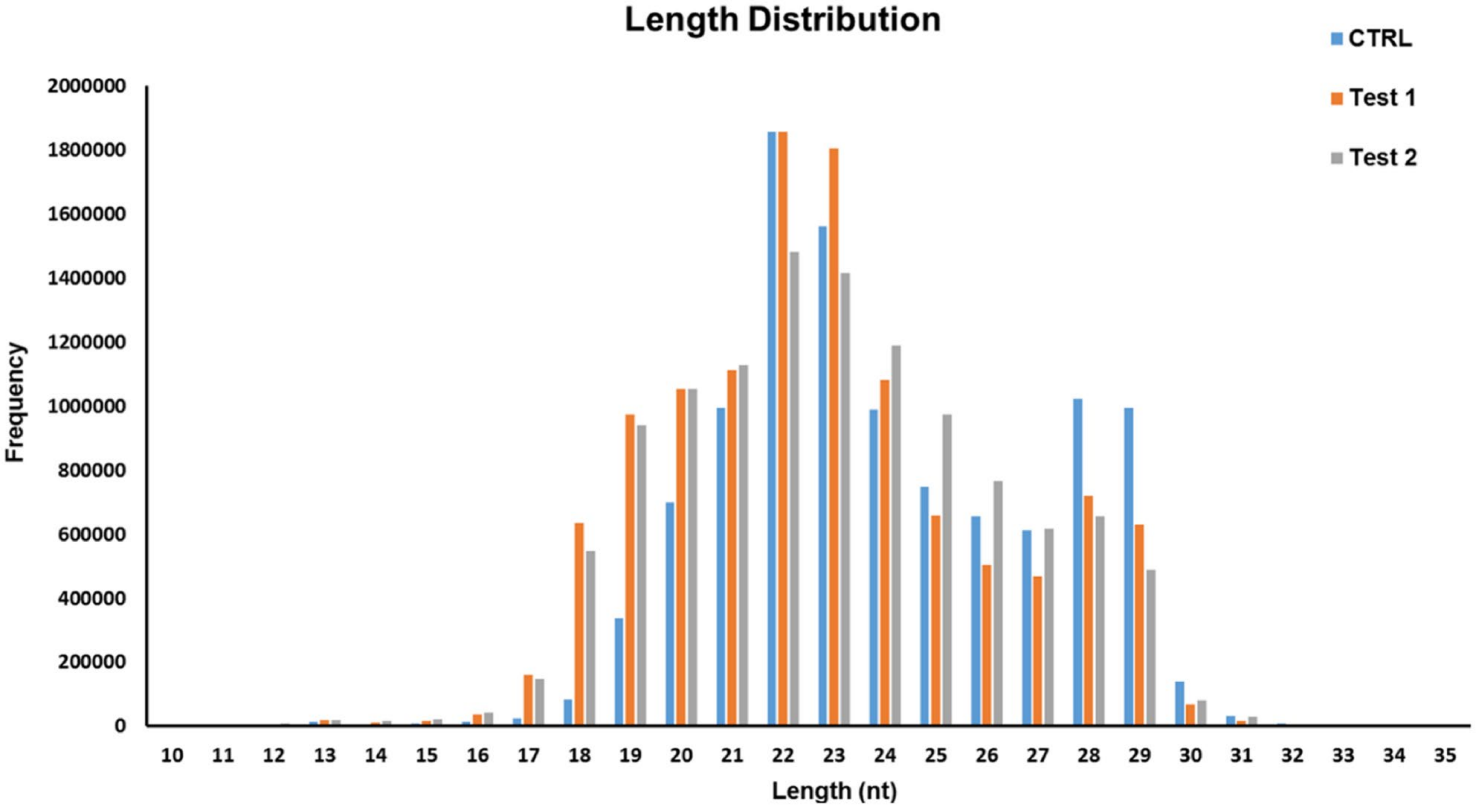
The distribution of size in sequenced small RNAs at all three libraries. Control: Small RNA library built from hUCB- MSCs under the control condition. Test 1 small RNA library derived from hUCB-MSCs treated with RA and Shh. Test 2: small RNA library obtained from Test 1 cells treated with a surviving factor, BDNF.

A total of 36,202,750 reads have been achieved from the three sRNA libraries (supplementary data 2). They were achieved by HiSeq 2500 (Illumina/Solexa sequencing platform), which was the next-generation sequencing technology. After filtering adaptors (low-quantity reads with 18 < poly(A) < 30 in length) the number of 33,714,015 reads were remained, which were group labeled based on their redundancy. Thus, the unique (non-redundant) sequences have been detected. The results showed that more than 50% of the sequences were 20 to 24-nt in length (Fig. 4). The 22-nt and 23-nt sRNAs were significantly more than the other sequences in the three libraries. The control and treatment groups had similar length distributions of small RNAs. The total number of sRNA reads derived from all three source libraries were identical, ranging from 12,019,568 and 11,836,522 to 11,138,629 reads in Test 1 (RA + /Shh +), Test 2 (RA-/Shh-), and Control groups. It indicated that the indexed sequencing strategy failed to generate a significant bias in the source libraries.

**Figure 5:**
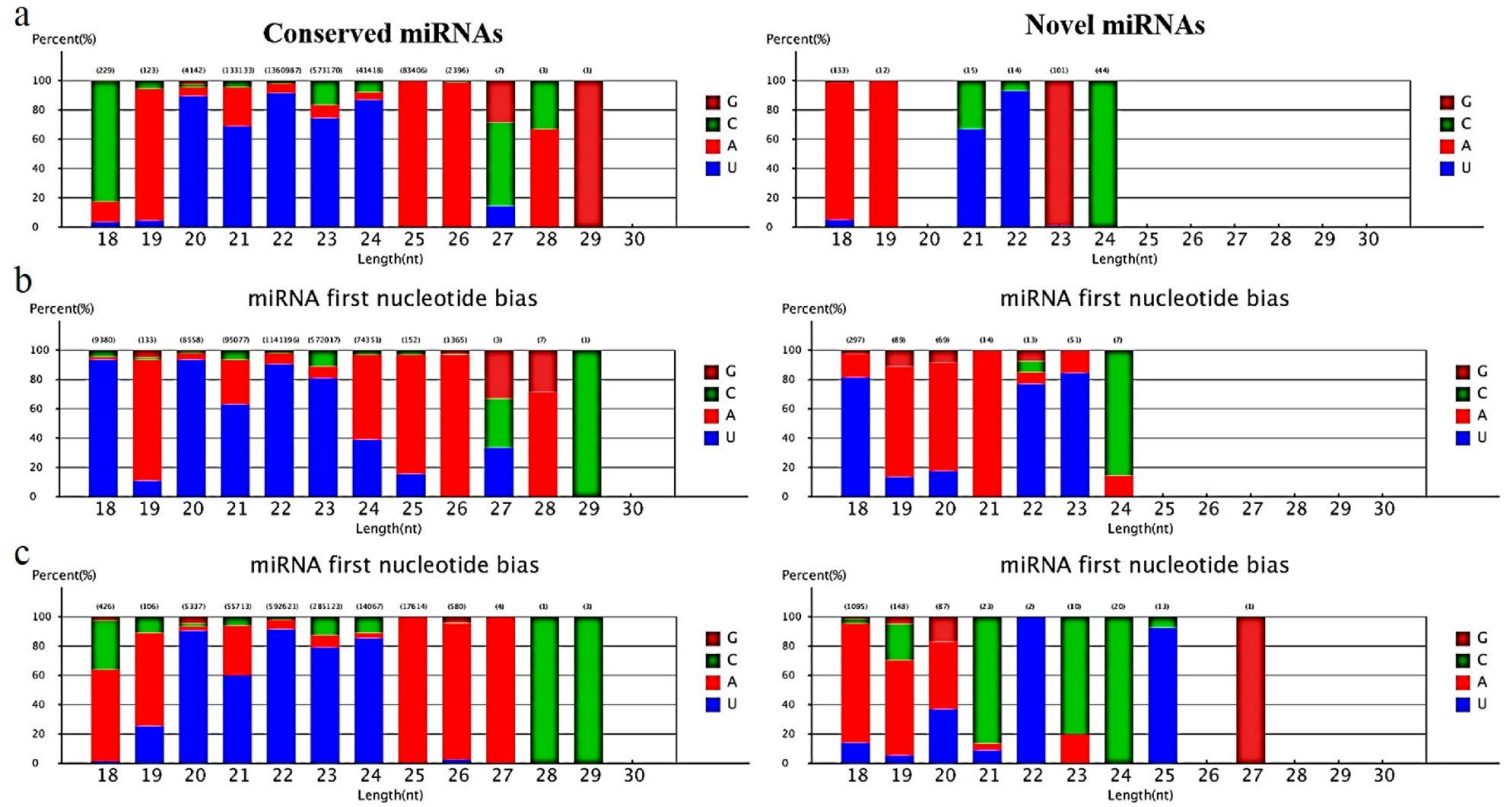
First nucleotide bias of the conserved and novel miRNAs at the three libraries. From up to down: Control, Test 1 and Test 2. Most miRNAs were started with 5ʹ-U and not with 5ʹ-G. This issue is in agreement with common mammalian miRNA sequence patterns.

In all three libraries, the unannotated sRNAs, rRNAs, miRNAs, and tRNAs have most of the reads in total small RNAs (supplementary data 3). Whereas, in the unique small RNA sequences, the unannotated sRNAs, rRNAs, exon-sense RNAs, and tRNAs are the most abundant reads. Also, the small RNAs were divided into various categories. This process was performed by conducting BLAST searches against Rfam. Noncoding RNAs (i.e., the unannotated RNAs, rRNAs, and tRNAs) were the most abundant total and unique small RNAs, respectively. In this study, the small RNA categories were similar in the investigated three libraries. Analyzing the first nucleotide (it was 18 to 23-nt long) of sRNAs suggested that most sRNAs, especially 20 and 22, were started by uridine (U) at their 5’-ends (Fig. 5).

### Identification of conserved miRNAs

**Figure 6:**
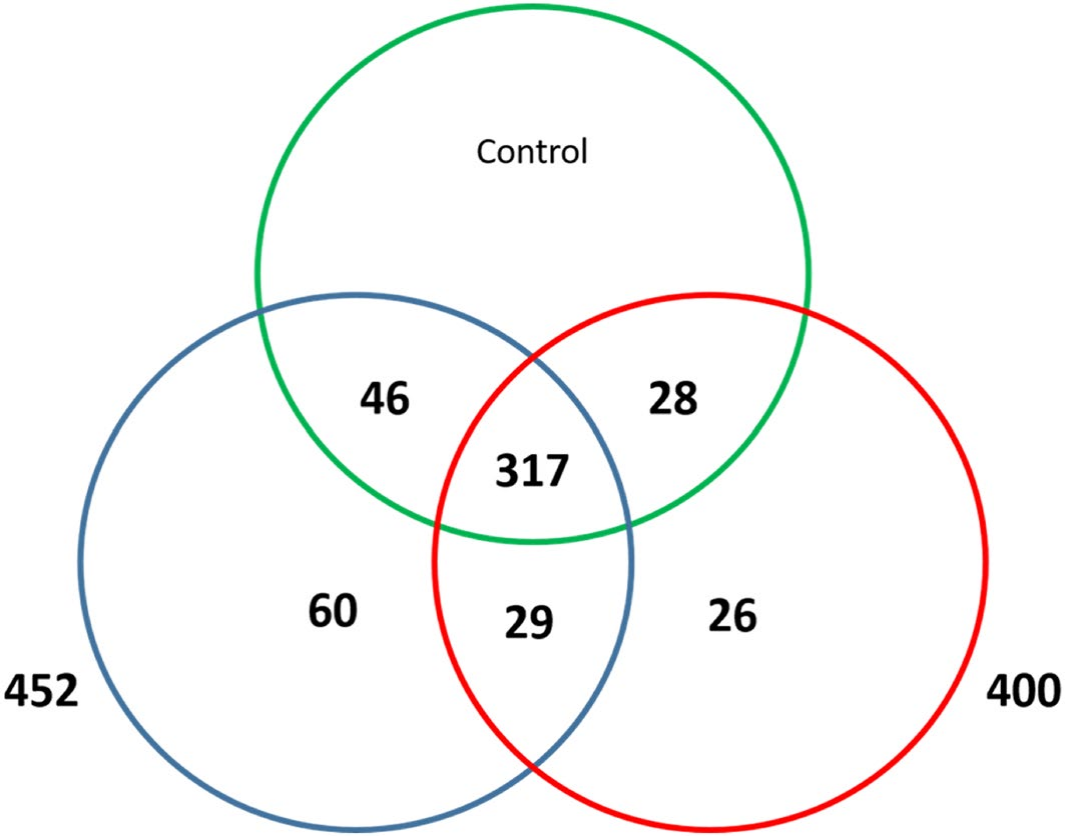
Total number of conserved miRNA families in test groups. Overall. 75, 60 and 26 miRNA families were only detected in Control(green), Test 1 (blue) and Test 2 (red), respectively.

In our experiment, it was possible to identify and characterize the conserved miRNAs. A majority of the conserved miRNAs were associated with 22-nt and 23-nt sequences. The 21-nt and 24-nt miRNAs were detected in several conserved miRNA families. In general, they were less frequent than the 22-nt and 23-nt miRNAs. The conserved miRNAs were detected in the Control, Test 1, and Test 2 libraries by searching all unique sRNA sequences and aligning them against the known miRNA database in miRBase 21. A total of 770, 749, and 659 miRNAs were found in the Control, Test 1, and Test 2 libraries, respectively. According to these miRNAs, the numbers of 127, 126, and 112 unique miRNAs were detected in each group, respectively. Some of the miRNAs demonstrated significantly low expression levels at the libraries with an actual read count of less than 10 (e.g., hsa-miR-98-3p, hsa-miR-200b-5p, and hsa-miR-150-5p). These low-expression miRNAs were not used for further expression analysis. The read counts were different between 328 known miRNAs. In these three libraries, the miRNAs belonged to 56 conserved families. Overall, the numbers of 466, 452, and 400 miRNA families were identified in the Control, Test 1, and Test 2 groups, respectively (Fig. 5). Several families (e.g., hsa-let-7, hsa-miR-21, and hsa-miR-30) were relatively abundant, while other families were less frequent. A total of 75, 60, and 26 miRNA families have only been detected in the Control, Test 1 (RA + /Shh +), and Test 2 (RA-/Shh-) groups, respectively (Fig. 6). Also, 317 families were common within these three libraries, while 336 miRNA families were common between Test 1 (RA + /Shh +) and Test 2 (RA-/Shh-) groups. As illustrated in Fig. 3, most miRNAs were started with 5ʹ-U and not with 5ʹ-G. This issue is in agreement with common mammalian miRNA sequence patterns.

### Identification of novel miRNAs

All unannotated small RNAs were searched against the human genome sequence to detect new miRNA sequences. For this purpose, strict criteria were used to detect the potential new miRNA loci by the mRDeep program. In this case, all precursors of the predicted new miRNAs could be folded into stem-loop structures. Then, the low-expression miRNAs (with an actual count of reads < 10) were eliminated. Thus, 77 potential novel miRNAs were predicted in the three libraries (Table 2). Accordingly, the numbers of 13, 24, and 27 unique sequences have been identified as novel miRNAs in the Control, Test 1 (RA + /Shh +), and Test 2 (RA-/Shh-) libraries, respectively. The novel miRNA sequences were 20 to 23-nt in length. The most abundant sequence was 21-nt reads.

**Table 2:** List of novel microRNAs and their consensus sequences.

Novel Mirna Name	miRDeep2 score	Estimated probability that the miRNA candidate is a true positive	Total read count	Consensus mature sequence	Precursor coordinate	Control	Test 2	Test 1
Novel miR1	1.6	0.71 ± 0.18	117	agagcuuaugagggagga	chr1:24,197,316..24197366: −	*		*
Novel miR2	1.8	0.71 ± 0.18	102	gcuuguugugauuccuccauuuu	chr5:180,528,922..180528988: +	*		
Novel miR3	2.3	0.85 ± 0.23	15	cuggacauguccuacgagcagcug	chr1:113,741,496..113741550: +	*	*	
Novel miR4	1.2	0.71 ± 0.18	12	aaaaugaaggacaagcaga	chr11:19,690,512..19690558: +	*	*	*
Novel miR5	1.5	0.71 ± 0.18	10	ugugugugugcuuguauaugu	chr19:7,687,944..7688018: −	*		
Novel miR6	− 2.3	0.68 ± 0.04	2	cccggcggcugugucuucaca	chr16:18,433,101..18433153: −	*		
Novel miR7	2.7	0.85 ± 0.23	12	ugacaacuauggaugagcucuc	chr15:81,134,335..81134391: +	*	*	*
Novel miR8	0.4	0.16 ± 0.17	8	auccgugauugggcucca	chr1:161,416,434..161416513: −	*		
Novel miR9	0.7	0.16 ± 0.17	8	ccuucggggugcugggcugcgggc	chr7:99,933,248..99933320:-	*		
Novel miR10	− 0.4	0.08 ± 0.12	6	ucucugggccugugucuu	chr19:46,142,264..46142328: −	*		
Novel miR11	4.3	0.91 ± 0.29	4	uuauccuccaguagacuaggga	chr8:99,405,894..99405952:-	*	*	
Novel miR12	1.8	0.71 ± 0.18	102	gcuuguugugauuccuccauuuu	chr5:180,524,407..180524473: −	*		
Novel miR13	− 2.3	0.68 ± 0.04	2	gugggggagaggcuguca	chr1:151,800,164..151800238: +	*		
Novel miR14	− 2.3	0.68 ± 0.04	2	cccggcggcugugucuucaca	chr16:16,466,468..16466520: +	*		
Novel miR15	3.3	0.93 ± 0.21	4	uuauccugcaucuguacaauca	chr15:33,036,884..33036937: +	*		
Novel miR16	0.7	0.16 ± 0.17	8	ccuucggggugcugggcugcgggc	chr7:72,476,837..72476909: +	*		
Novel miR17	1.3	0.43 ± 0.18	104	uuugcuugugagauuuug	chr1:173,551,238..173551315: +		*	*
Novel miR18	1.4	0.43 ± 0.18	69	uuugcuuggguuguuuca	chr16:25,097,149..25097193: −		*	*
Novel miR19	0.5	0.00 ± 0.00	80	aaaggaggaaaaacuguugu	chr4:152,430,080..152430124: −		*	*
Novel miR20	0.6	0.00 ± 0.00	24	aagacauuuuaauagaua	chr18:27,312,173..27312218: +		*	*
Novel miR21	0	0.00 ± 0.00	22	ucuacaacugaaacuggc	chr10:124,756,338..124756390: +		*	
Novel miR22	1.2	0.43 ± 0.18	91	ugagugugugugugcgagugugu	chr2:157,746,696..157746770: +		*	
Novel miR23	2.2	0.69 ± 0.30	16	aacgaucaggaacaagcu	chr14:102,551,934..102551988: −		*	*
Novel miR24	1.4	0.43 ± 0.18	17	ucugugucuggaccaaga	chr19:15,384,987..15385039: −		*	*
Novel miR25	0	0.00 ± 0.00	15	uuugcugcuaaauucauu	chr12:80,085,550..80085598: −		*	
Novel miR26	1.3	0.43 ± 0.18	12	ugaguguacuugcccagacu	chr7:94,141,301..94141369: +		*	
Novel miR27	1.3	0.43 ± 0.18	10	agccuugucaucucugagcu	chr22:50,836,017..50836085: +		*	
Novel miR28	1.8	0.43 ± 0.18	15	aaaagggggcugagguggagg	chr11:122,022,809..122022867: −		*	
Novel miR29	0.7	0.16 ± 0.17	8	ccuucggggugcugggcugcgggc	chr7:74,307,106..74307178: +	*		
Novel miR30	0.4	0.00 ± 0.00	10	ucuagauugccuuucucc	chr2:113,759,179..113759218: −		*	
Novel miR31	1.7	0.43 ± 0.18	12	ugagugugugggugugggugugu	chr9:137,651,227..137651303: −		*	
Novel miR32	0.3	0.00 ± 0.00	12	ucuggauaugaucuuagc	chr13:105,353,355..105353432: +		*	*
Novel miR33	0.4	0.00 ± 0.00	8	guuugaauccugucucca	chr1:29,210,270..29210333: −		*	
Novel miR34	0.3	0.00 ± 0.00	10	aaagcaaauguugggugaacggc	chr12:64,217,443..64217508: +		*	
Novel miR35	0.4	0.00 ± 0.00	8	aggaccaucacaggcuga	chr16:88,921,058..88921111: +		*	
Novel miR36	1.1	0.43 ± 0.18	12	aagaauuguugaauucuu	chr15:33,746,138..33746177: +		*	
Novel miR37	0.2	0.00 ± 0.00	9	gucaugggcuggguuuuacu	chr17:22,029,965..22030036: −		*	
Novel miR38	0.4	0.00 ± 0.00	13	uuuguugguuagguaguug	chr5:134,260,620..134260691: +		*	
Novel miR39	0	0.00 ± 0.00	9	uauugauuucacggaggauggug	chr9:34,999,196..34999250: +		*	
Novel miR40	0.1	0.00 ± 0.00	10	ggugcaucuguaauuucuc	chr5:162,317,313..162317352: +		*	*
Novel miR41	1.7	0.43 ± 0.18	8	ucuggggcccugggcagaccucc	chr13:110,889,374..110889435: −		*	
Novel miR42	0.7	0.16 ± 0.17	8	ccuucggggugcugggcugcgggc	chr7:74,987,986..74988058:-	*		
Novel miR43	0.7	0.00 ± 0.00	10	gugaagaacauugaugaug	chr1:159,729,047..159729099: +		*	
Novel miR44	1.2	0.43 ± 0.18	11	caguggcuguuucuuugu	chr5:156,191,915..156191954: −		*	
Novel miR45	− 2	0.00 ± 0.00	2	acuggacuuggagucagaagg	chr5:149,112,393..149112451: +		*	
Novel miR46	− 9.9	0.00 ± 0.00	3	acuggacuuggagucagaagg	chr3:32,027,758..32027820: −		*	
Novel miR47	2.6	0.69 ± 0.30	2	uuuuugucaguacauguuaaug	chr10:5,729,070..5729133: +		*	
Novel miR48	1.7	0.37 ± 0.15	10	ggaggagcuggggcuggg	chr10:134,258,407..134258454: −			*
Novel miR49	− 9.4	0.00 ± 0.00	1	ucguguggaucuauacuucuaag	chr10:15,171,670..15171743: −		*	
Novel miR50	#######	0.00 ± 0.00	1	gagaguauagaauuggaggcag	chr17:62,646,615..62646674: −		*	
Novel miR51	− 2.7	0.30 ± 0.04	1	agcuaaaugugugcugggacau	chr11:28,078,376..28078433: −		*	
Novel miR52	1.1	0.37 ± 0.15	22	uuuguagguaaauucugc	chr4:185,114,901..185114956: −			*
Novel miR53	2.7	0.79 ± 0.13	36	cuucucgaggcuccggcggcu	chr17:80,212,581..80212671: −			*
Novel miR54	1.8	0.37 ± 0.15	10	uggggcggagcgucuggaag	chr16:2,186,120..2186204: +			*
Novel miR55	2	0.79 ± 0.13	16	cagcuggggaaacugaggcc	chr2:192,260,003..192260070: +			*
Novel miR56	2.1	0.79 ± 0.13	10	uggggcggagcgucuggaag	chr16:15,248,807..15248869: +			*
Novel miR57	0.5	0.00 ± 0.00	31	cagauuuuggauuucauuu	chr2:101,596,388..101596462: −			*
Novel miR58	1.5	0.37 ± 0.15	36	caguggcuguuucuuugu	chr5:156,191,915..156191954: −			*
Novel miR59	1	0.37 ± 0.15	12	aagaucauuuagaaccac	chr9:109,676,406..109676442: +			*
Novel miR60	2.6	0.79 ± 0.13	12	cgcgcgcguguguggugug	chr5:122,403,765..122403832: +			*
Novel miR61	1	0.37 ± 0.15	12	aauuaguuauugaucgug	chr15:97,192,137..97192186: −			*
Novel miR62	2.4	0.79 ± 0.13	17	aucauggagaaggcuggggc	chr1:117,257,061..117257151: −			*
Novel miR63	2.2	0.79 ± 0.13	10	uggggcggagcgucuggaag	chr16:2,186,078..2186140: +			*
Novel miR64	1.6	0.37 ± 0.15	10	ggaggagcuggggcuggg	chr9:140,202,744..140202804: −			*
Novel miR65	1.3	0.37 ± 0.15	19	cuucagccggaauuacuuu	chr12:114,773,141..114773216: −			*
Novel miR66	1.60E + 01	0.86 ± 0.24	38	caaguggaggaagaagcgaaugcg	chr20:21,735,891..21735940: +			*
Novel miR67	1.8	0.37 ± 0.15	9	cgguggcggcggcggagg	chr2:48,132,674..48132748: +			*
Novel miR68	0.3	0.00 ± 0.00	12	ugaguuugcuugugagau	chr2:206,077,134..206077197: +			*
Novel miR69	1.4	0.37 ± 0.15	9	cgguggcggcggcggagg	chr2:48,132,624..48132692: +			*
Novel miR70	1.7	0.37 ± 0.15	14	ucuugggcgggguuggggg	chr2:22,368,144..22368192: −			*
Novel miR71	0.6	0.00 ± 0.00	8	cuguguuuaucggagugggggug	chr19:5,043,225..5043298: +			*
Novel miR72	0.4	0.00 ± 0.00	23	aguuuguugguuagguaguu	chr5:134,260,618..134260693: +			*
Novel miR73	1.50E + 01	0.86 ± 0.24	38	caaguggaggaagaagcgaaugcg	chr7:137,198,588..137198637: +			*
Novel miR74	4.8	0.86 ± 0.21	5	uuauccuccaguagacuaggg	chr8:99,405,895..99405952: −			*
Novel miR75	2.6	0.79 ± 0.13	2	ccucugggagcuauggguuggcaug	chr20:2,636,889..2636963: +			*
Novel miR76	− 6.6	0.00 ± 0.00	1	ucccuguccuccaggagcuc	chr7:1,062,594..1062648: −			*
Novel miR77	− 6.6	0.00 ± 0.00	1	ucccuguccuccaggagcuc	chr7:1,062,628..1062674: −			*

### Prediction and annotation of miRNA target genes

In this section, the putative targets were predicted for the conserved and novel miRNAs by the miRanda program. Then, the functions of the conserved and novel miRNAs have been predicted. The Gene Ontology (GO) analysis has been performed to evaluate the possible functions of the miRNA target genes. The detected miRNAs targets have been predicted based on the human genome sequence. Many target positions have been predicted for the known conserved miRNAs in the sequencing libraries. A total of 621, 624, and 562 potential target genes were identified for the conserved miRNAs in the Control, Test 1 (RA + /Shh +), and Test 2 (RA-/Shh-) groups. Also, 16, 39, and 38 potential target genes have been identified for the novel miRNAs in the Control, Test 1 (RA + /Shh +), and Test 2 (RA-/Shh-) groups, respectively. This information is provided in Table 3. Besides, Table 4 gives the number of miRNAs with different expressions and their targets. Many miRNAs had more than one predicted target site so that the targets predicted for the conserved and novel miRNAs were cellular components, biological processes, and molecular functions. Also, the target genes were categorized into 23, 17, and 14 classes as a cellular component, biological process, and molecular function, respectively. The most over-represented GO terms in the biological process were comprised of cellular process, single-organism process, and metabolic process, respectively. It means that most of the target genes play significant roles in this category. The most over-expressed GO terms in the cellular component were related to the cell, cell parts, and organelle classes. A majority of the miRNAs categorized in the molecular function class had binding and catalytic activities (supplementary data 4). In this study, the KEGG pathway database has been employed to understand the high-level functions and pathways in the cells. These were derived from the generated miRNA information by sequencing. The KEGG pathway analysis has been accomplished to explore the biological interpretation of the target genes. A total of 308 different pathways were found for the conserved and novel miRNA target genes. Among them, six pathways were chosen as the model signaling pathways. These pathways were cholinergic synapse, Hedgehog, JAK-STAT, axon guidance, TGF-beta, and MAPK signaling pathways. In this case, these selected six pathways had a significant effect on motor neuron differentiation in human development. Table 5 provides the most abundant target genes annotated by these six selected pathways. These target genes have differentially been expressed in the Control versus Test 1 (RA + /Shh +) and Control versus Test 2 (RA-/Shh-).

**Table 3:** Numbers of mirRNA targets in the three libraries of Control (CTRL), Test 1 and Test 2.

	Groups	miRNA number	Target gene number	miRNA:target number
Conserved miRNA	CTRL	621	36,223	404,556
	Test 1	624	36,265	412,943
	Test 2	562	36,135	374,366
Novel miRNA	CTRL	16	28,183	42,445
	Test 1	39	21,257	58,724
	Test 2	38	19,829	56,756

**Table 4:** Numbers of differentially expressed conserved and novel miRNAs and their targets among the three libraries of Control (CTRL), Test 1 and Test 2.

	Groups	miRNA number	Target gene number	miRNA:Target number
Conserved miRNA	CTRL* Test1	99	28,372	63,206
	CTRL*Test 2	107	28,470	66,928
	Test1*Test 2	211	33,028	122,298
Novel mirna	CTRL*Test 1	31	26,786	54,261
	CTRL*Test 2	34	27,762	58,296
	Test 1*Test 2	40	21,314	60,688

**Table 5:** The most abundant target genes annotated with the 6 selected pathways differentially expressed in control versus Test1, Control versus Test 2 and Test 1 versus Test 2 groups.

Pathway	Groups	Target Genes with Pathway Annotation (Known)	*P*- value (*:*P* < 0.05)	Target Genes with Pathway Annotation (Novel)	*P*- value (*:*P* < 0.05)
Cholinergic Synapse	Ctrl-vs-T1	245(1.07%)	0.449	235(1.2%)	*
	**Ctrl-vs-T2**	**259(1.13%)**	*	**233(1.15%)**	*
	T1-vs-T2	294(1.11%)	*	186(1.13%)	0.094
Axon Guidance	Ctrl-vs-T1	444(1.93%)	*	362(1.85%)	0.312
	Ctrl-vs-T2	449(1.96%)	0.213	358(1.77%)	0.833
	T1-vs-T2	495(1.87%)	*	289(1.76%)	0.792
Hedgehog Signaling Pathway	Ctrl-vs-T1	94(0.41%)	0.189	83(0.42%)	0.107
	**Ctrl-vs-T2**	**99(0.43%)**	*	**88(0.43%)**	*
	T1-vs-T2	104((0.39%)	0.415	79(0.48%)	*
JAK- STAT Signaling PAthway	Ctrl-vs-T1	284(1.24%)	0.441	245(1.25%)	0.323
	**Ctrl-vs-T2**	**300(1.31%)**	*	**266(1.31%)**	*
	T1-vs-T2	325(1.23%)	0.501	227(1.39%)	*
TGF- beta Signaling Pathway	Ctrl-vs-T1	203(0.88%)	*	159(0.81%)	0.477
	Ctrl-vs-T2	190(0.83)	0.271	158(0.78%)	0.790
	T1-vs-T2	218(0.83%)	0.247	134(0.82%)	0.445
MAPK Signaling Pathway	Ctrl-vs-T1	722(3.14%)	0.701	0.653(3.34%)	*
	Ctrl-vs-T2	719(3.14%)	0.725	676(3.34%)	*
	T1-vs-T2	823(3.11%)	0.911	542(3.31%)	0.079

Data were extracted after copyright permission from KEGG database and referenced in discussion.

Significant values are in [bold].

### Differential expression of miRNAs in different libraries

**Figure 7:**
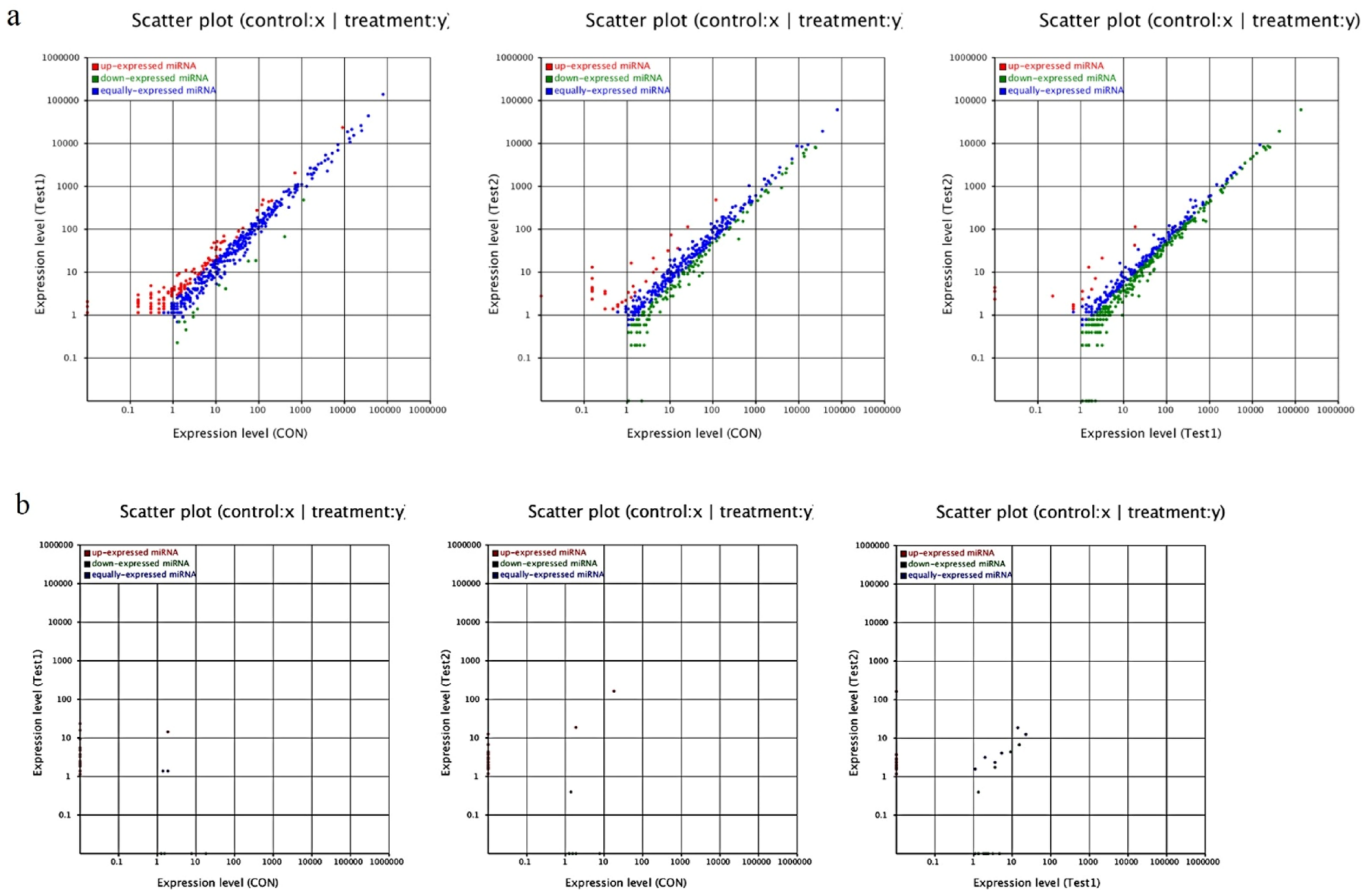
Differential expression patterns of miRNAs between test groups. According to the number of reads counted by high-throughput sequencing, a comparison was drawn between the expression levels of conserved (**a**) and new (**b**) miRNAs at the three libraries. Scatter plots indicate the frequency of conserved and novel miRNAs in Test1 versus Control (left), Test2 versus Control(middle) and Test 2 versus Test 1 (right).

The data between groups have been analyzed to find the miRNAs. The results showed significant up or down-regulation in comparison with the control group. The reads of the known and novel miRNA sequences were compared in the three libraries to find the differentially regulated miRNAs in the Control, Test 1 (RA + /Shh +), and Test 2 (RA-/Shh-) groups. In these libraries, the expression levels of the conserved (Fig. 7a) and new (Fig. 7b) miRNAs were compared. This procedure was performed by drawing on the number of reads counted and high-throughput sequencing. The number of specific miRNAs was significantly different between these three libraries.

Overall, 111, 128, and 239 conserved miRNAs had different expressions in the Control versus Test 1 (RA + /Shh +), Control versus Test 2 (RA-/Shh-), and Test 1 (RA + /Shh +) versus Test 2 (RA-/Shh-), respectively. A comparison between the miRNA expression profiles in Test 1 group (RA/Shh +) and the Control group showed that ten miRNAs were down-regulated. For example, hsa-miR-449c-5p, hsa-miR-1249-3p, hsa-mir-9-5p, and mir-324 were up-regulated. While hsa-miR-335-3p and hsa-miR-335-5p were significantly down-regulated.

A comparison between the miRNA expression profiles in Test 2 (RA/Shh-) and the Control groups demonstrated that 106 miRNAs were down-regulated. For example, miR-let7b, miR-137, hsa-miR-149-3p, hsa-miR-3940-3p, and hsa-miR-6511-5p were down-regulated, whereas hsa-miR-619-5p and hsa-miR-3687 were significantly up-regulated. A comparison between the expression profiles in Test 1 group and Test 2 group revealed that 230 miRNAs (e.g., hsa-mir-9-5p) were down-regulated in Test 2. Also, nine miRNAs (e.g., has-miR-612, has-miR-4683, and has-miR-3687) were up-regulated.

A total of 32, 35, and 41 novel miRNAs showed different expression levels in the Control versus Test 1, Control versus Test 2, and Test 1 versus Test 2, respectively. A comparison between the miRNA expression profiles in Test 1 and Control groups showed that 27 miRNAs were up-regulated. For example, novel-mir-17, 18, and 20 were up-regulated, whereas novel-miR-1 and novel-miR-2 were significantly down-regulated.

A comparison between the miRNA expression profiles in Test 2 and Control groups suggested that 30 miRNAs were up-regulated. For example, novel-miR-2, 3, and 6 were down-regulated, whereas novel-miR-18, 19, and 20 were significantly up-regulated.

Also, the expression profile of the Test 1 group has been compared with the Test 2 group. The results revealed that 21 miRNAs were down-regulated, while 20 others were up-regulated. The miRNAs such as novel-miR-1, novel-miR-52, and novel-miR-53 were up-regulated (Table 6).

**Table 6:** Differential expression in candid conserved microRNAs between Control, Test 1 (RA + /Shh +) and Test 2 (RA-/Shh-) groups.

miR_name	CON1-total-reads	Test1-total-reads	CON1-expressed	Test1-expressed	CON1-std	Test1-std	Fold-change(log2 Test1/CON1)	*p*-value	Sig-lable
hsa-miR-9-5p	6,353,410	4,421,513	10	21	1.574	4.7495	1.59334	0.002839	**
hsa-miR-324-5p	6,353,410	4,421,513	2	11	0.3148	2.4878	2.982363	0.001605	**

miR_name	CON1-total-reads	Test2-total-reads	CON1-expressed	Test2-expressed	CON1-std	Test2-std	Fold-change(log2 Test2/CON1)	*p*-value	Sig-lable
hsa-let-7b-5p	6,353,410	5,146,805	159,024	41,106	25,029.71	7986.702	− 1.64797	0	**
hsa-miR-137	6,353,410	5,146,805	308	115	48.4779	22.344	− 1.11744	1.11E-13	**

miR_name	Test1-total-reads	Test2-total-reads	Test1-expressed	Test2-expressed	Test1-std	Test2-std	Fold-change(log2 Test2/Test1)	*p*-value	Sig-lable
hsa-miR-9-5p	4,421,513	5,146,805	21	8	4.7495	1.5544	− 1.61142	0.004807	**
hsa-miR-324-5p	4,421,513	5,146,805	11	3	2.4878	0.5829	− 2.09355	0.016434	*
hsa-let-7b-5p	4,421,513	5,146,805	114,725	41,106	25,947	7986.702	− 1.6999	0	**

The analysis of miRNA sequencing revealed a significant up-regulation of mir- 9-5p and mir-324-5p in Test 1 (RA + /Shh +); and down- regulation of mir-137 and let-7b in Test 2 (RA-/Shh-) when the results were compared with the control.

### The qRT-PCR analysis of miRNA expression

**Figure 8:**
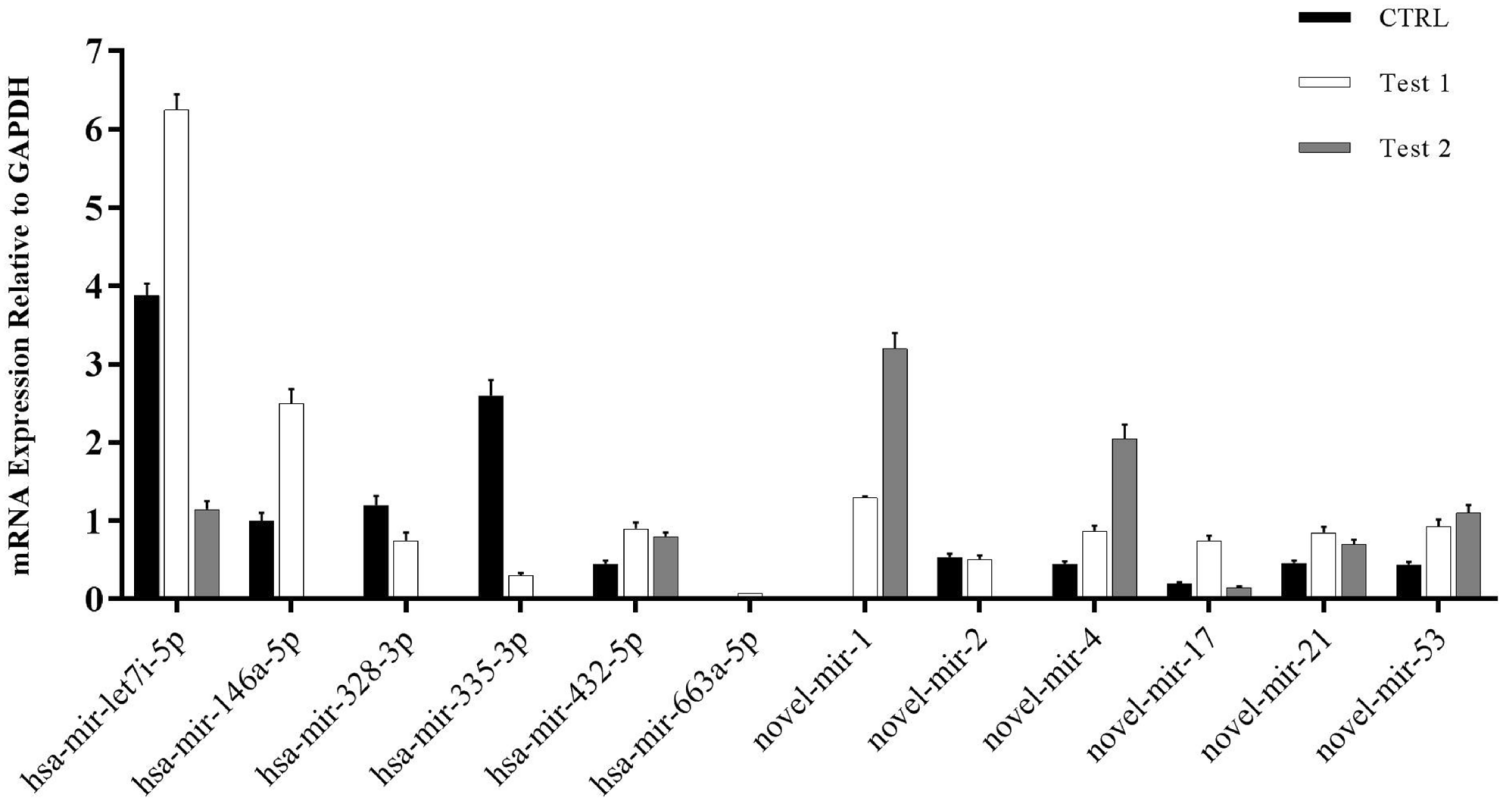
The qRT-PCR analysis of miRNA expression. The qRT-PCR technique was used to verify and measure the expression levels of six conserved miRNAs and six novel miRNAs in Control, Test 1 and Test 2 groups. The qRT-PCR results revealed that all tested miRNAs were detected similar to what illustrated by the performed high-throughput sequencing analysis. However, the expression levels of hsa-miR-432-5p, hsa-miR-335-3p, novel-miR-2, and novel-miR-21 were different from the achieved sequencing outcomes. Values are expressed as the mean ± SEM.

The real-time quantitative PCR (qRT-PCR) analysis has been completed to verify and measure the differential expression levels of miRNAs. The proposed known and novel miRNAs exhibited significant expression differences in the three libraries. The expression levels of the six conserved miRNAs and six new miRNAs in the Control, Test 1, and Test 2 groups were validated and measured by the qRT-PCR technique. All randomly chosen miRNAs were identified by high-throughput sequencing in the Control, Test 1, and Test 2 groups. According to the qRT-PCR analysis, all tested miRNAs have been identified in the groups (Fig. 8). These miRNAs had different expression levels. The results of the qRT-PCR demonstrated that the expression levels of the new miRNAs were different in comparison with the control and the test groups. These novel miRNAs have a Ct value, ranging from 34.56 ± 3.24 to 34.65 ± 1.68. The qRT-PCR results revealed that hsa-miR-let7i-5p, hsa-miR-146a-5p, hsa-miR-328-3p, hsa-miR-663a-5p, novel-miR-1, novel-miR-4, as well as novel-miR-17, and novel-miR-53 have expression patterns, which are comparable to those illustrated by the performed high-throughput sequencing analysis. The expression levels of hsa-miR-432-5p, hsa-miR-335-3p, novel-miR-2, and novel-miR-21 were not matched with the achieved sequencing outcomes. This issue was due to the sequencing error or other unknown reasons (Fig. 8).

## Discussion

Since motor neuron-related disorders and diseases are dramatically common worldwide, the importance of stem cell-derived motor neurons as an in vitro model used for drug screening in neuromuscular disorders is not evitable. Many types of researches have been performed to provide motor neurons via various stem cells, including MSCs and iPSCs. These approaches have been developed for use in neural injury modeling and drug screening. Although both miRNA-based differentiation of hiPSCs and mesenchymal stem cells into neural lineages have been reported, there is still little knowledge regarding the role of miRNAs in regulating motor neuron differentiation.

The purpose of this study was to explore miRNA profiles of human CB-MSCs during differentiation into motor neurons in the presence of two morphogens, naming RA and Shh. These morphogens were responsible for motor neuron generation. After differentiation induction, the cells expressed motor neuron-related markers (i.e., Islet-1, SMI-32, and ChAT) at the level of mRNA and protein. This procedure was performed using immunocytochemistry and flow cytometry. In this study, three small RNA libraries have been provided to investigate the miRNA profile of differentiating cells. These libraries were derived through the following groups: (1) The cells treated by RA and Shh: Test1 (RA + /Shh +), (2) The cells after removing of RA and Shh from the medium: Test2 (RA-/Shh-), and (3) The Control (non-treated human UCB-MSCs). The miRNA sequencing revealed a significant up-regulation in the expression of miR-9-5p and miR-324-5p at the early stage of differentiation. But the expressions of miR-137 and let-7b were down-regulated at the next stage of the differentiation in Test 2. They were all responsible for neuron and motor neuron differentiation and suppression of proliferation in neural progenitor cells. Also, we could find some novel miRNAs involved in cholinergic, JAK-STAT, Hedgehog, and MAPK signaling pathways during differentiation.

The miRNAs have been known to be the most remarkable regulatory molecule involved in neurogenesis^21^. Despite many studies that suggested the inhibition of miRNA biogenesis results in inappropriate neuro-regulation, the biology of miRNAs during neuronal differentiation has not properly been understood. Indeed, there is little knowledge regarding the role of miRNAs in the process of motor neuron development. The previous studies have failed to capture the dynamic profile of miRNA expression during neurogenesis^22^. Therefore, this study evaluates the miRNA profile of differentiating hUCB-MSCs during differentiation into motor neuron-like cells. This procedure was accomplished by applying RA and Shh and developing a two-step protocol. At the first step of the induction protocol, the RA and Shh were employed synergistically to mimic the developmental pattern of the neural tube through the rostrocaudal (by RA) and dorsoventral (by Shh) axes. Then, RA and Shh were removed, and the cells were incubated with a neurotrophic factor, naming BDNF. In the previous study, the authors assessed the differentiation potential of human chorion-derived mesenchymal stem cells into motor neuron-like cells in two- and three-dimensional culture systems. Similarly, it is confirmed that chorion-derived mesenchymal stem cells conveniently access various types of cells, which can differentiate into motor neuron-like cells in the presence of RA and Shh in a two-dimensional culture system and on an electro spun gelatin scaffold^11^.

In the present study, the hsa-miR-9-5p expression could be detected after induction with RA and Shh in Test 1 group. However, the expression of this miRNA significantly dropped in Test 2 group. The miR-9 was known to be a highly conserved miRNA in the central nervous system. This miRNA regulates the normal development of the brain^23^. In 2016, Sim et al.^24^ studied the brain-enriched miRNA (i.e., miR-9-3p), which regulates synaptic plasticity and memory. They demonstrated that miR-9-3p and miR-9-5p are involved in the normal development of the central nervous system. This procedure was carried out by striking a balance between differentiation and self-renewal of neural stem cells. The miR-9 affects the TLX expression in the neural stem cells^25^. The TLX expression is constrained during the differentiation of neural stem cells. But the miR-9 expression increases at the same time. Also, the miR-9 controls the differentiation of early motor neurons into mature motor neurons by regulating the expression of the Onecut transcription factor. Moreover, the Onecut supports the expression of Islet-1 and the lateral motor column LMCms. The interaction of Islet-1 and another transcription factor (i.e., Lhx1) supports the formation of the lateral motor column^26–28^.

In 2017, Schaller et al.^29^ demonstrated that the development of the Lhx1p lateral motor column depends on the RA secreted from lateral motor column LMCms. Also, Otaegi et al.^27^ confirmed that regulatory networks are currently unknown within the downstream of RA and the upstream of Islet-1/Lhx1. The FoxP1 is another transcription factor involved in preganglionic and lateral motor neurons. The miR-9 controls motor neuron formation by regulating the level of FoxP1 expression.

The results indicated a significant down-regulation of miR-let7b in Test 2 group compared to the Control group. The down-regulation of miR-let7b supports the proliferation of neural stem cells. This miRNA targets TLX (a nuclear receptor and its effector cyclinD1) to control the proliferation and differentiation of neural stem cells. In 2014, Patterson et al.^28^ expressed that miR-let7b reduces the self-renewal properties of the neural progenitor cells in the brain of old adults. Indeed, miR-let7b controls the developmental fate of the neural progenitor cells into neurons or glial cells. It is performed by regulating the expression of HMGA2 and HES5 in the notch signaling pathway.

In the Test 1 group, the up-regulation of miR-324-5p could be identified in the presence of Shh as an inducing factor. This miRNA targets Gli-1 as a member of the Shh signaling pathway^30^.

The findings showed a significant down-regulation for the miR-137 expression in the Test 2 group by removing the RA and Shh. According to previous reports, miR-137 regulates the maturation of neurons. The down-regulation of this miRNA supports the morphogenesis of dendrites, cellular phenotypes, and the development of the spinal cord and early neural cells. The miR-137 regulates the ubiquitin ligase expression, called Mind Bomb-1^31^. Also, it targets H3K27 in EZH2 methyltransferase to control the proliferation and differentiation of neural progenitor cells in adults. It seems that miR-137 is involved in regulating the maturation of neurogenesis in the hippocampus. Also, it was managed to detect the expression of miR-132 in Test 2. This miRNA is expressed in neurons, but it was not in astrocytes or neural progenitor cells. Similar to miR-137, miR-132 is prevalent in dendrites. Unlike miR-137, miR-132 reduces the volume of the spine^32^.

In this study, some novel miRNAs were exclusively detected in one group or shared between other groups. For example, the miRs-3, 8, and 13 were expressed in the Control, Test 1, and Test 2 groups, respectively. A total of eight miRNAs were common only in Test 1 and Test 2 groups.

Also, we considered four candidate signaling pathways involved in neurogenesis, including the acetylcholine signaling pathway, Hedgehog signaling pathways, JAK-STAT signaling pathway, and MAPK pathway. After neural induction in Test 1 and Test 2 groups compared to the Control group, the results indicated the expression of miRNAs involved in the acetylcholine pathway. The novel miRNAs related to Shh signaling pathway were up-regulated in Test 1 group. It was confirmed by comparing the achieved results with the Test 2 group. This issue was probably due to the presence of Shh in the induction medium of the Test 1 group.

In the JAK-STAT signaling pathway, some novel miRNAs were up-regulated in Test 2 group. It was confirmed by comparing the attained results with the Control and Test 1 groups.

In the MAPK pathway, some novel miRNAs were found in test groups. It was confirmed by comparing the outcomes with the non-treated mesenchymal stem cells.

## Conclusions

This study revealed that CB-MSCs are potent to express the motor neuron-related markers at the mRNA level and the proteins in the presence of Shh and RA. On the other hand, the analysis of the miRNA profile of motor neuron-like cells showed a significant up-regulation in the expression of miR-9, miR-let7b, miR-137, and miR-324-5p, which are involved in the neural differentiation and neuron/motor neuron maturation. The miR-9-5p and miR-324-5p (they have roles in neural cell and motor neuron differentiation) were up-regulated at the early stage of differentiation. But the expressions of miR-137 and miR-let-7b (considering cell proliferation regulatory roles) were down-regulated at the next stage of differentiation. Moreover, some novel miRNAs related to the Cholinergic, Hedgehog, MAPK, and JAK-STAT signaling pathways were detected in the test groups. However, further studies are still necessary to validate their functions in the mechanisms related to motor neuron differentiation and maturation.

## Supplementary Information

Supplementary Information.
