# Supplementary material for: Large-scale analysis of MicroRNA expression in motor neuron-like cells derived from human umbilical cord blood mesenchymal stem cells
Source: Sci Rep. 2022 Apr 7;12:5894. doi: 10.1038/s41598-022-09368-6 (PMC8991218; doi:10.1038/s41598-022-09368-6)
Supplement: Supplementary file 1 — Supplementary Information. [file 41598_2022_9368_MOESM1_ESM.docx]

**Supplementary data 1a.** Primer sequences for each gene using the qRT-PCR.

| Gene | Sequence | Accession Number |
| --- | --- | --- |
| Nestin | F TCCAGGAACGGAAAATCAAG  R GCCTCCTCATCCCCTACTTC | NM_006617.1 |
| Hb-9 | F AGCACCAGTTCAAGCTCAACA  R ACCAAATCTTCACCTGGGTCTC | NM_005515.3 |
| Islet-1 | F ATATCAGGTTGTACGGGATCAAATG  R CACGCATCACGAAGTCGTTC | NM_002202.2 |
| ChAT | F GCA GGAGAAGACAGCCAACT  R TGCAAACCTCAGCTGGTCAT | NM_020549.4 |
| SMI-32 | F CAGAGCTGGAGGCACTGAAA  R CTGCTGAATGGCTTCCTGGT | NM_021076.3 |
| GAPDH | F CTCATTTCCTGGTATGACAAC  R CTTCCTCTTGTGCTCTTGCT | NM_002046.5 |

**Supplementary data 1b.** List of sequences of primers used for data validation.

| ­­­miRNA | Primer Type | | Primer Sequences |
| --- | --- | --- | --- |
| hsa-let-7i-5p | RT | CTCAACTGGTGTCGTGGAGTCCGGCAATTCAGTTGAGAACAGCAC | |
| hsa-mir-146a-5p | RT | CTCAACTGGTGTCGTGGAGTCCGGCAATTCAGTTGAGAACCCATG | |
| hsa-mir-328-3p | RT | CTCAACTGGTGTCGTGGAGTCCGGCAATTCAGTTGAGACGGAAGG | |
| hsa-mir-335-3p | RT | CTCAACTGGTGTCGTGGAGTCCGGCAATTCAGTTGAGGGTCAGGA | |
| hsa-mir-432-5p | RT | CTCAACTGGTGTCGTGGAGTCCGGCAATTCAGTTGAGCCACCCAA | |
| hsa-mir-663a-5p | RT | CTCAACTGGTGTCGTGGAGTCCGGCAATTCAGTTGAGGCGGTCCC | |
| novel_mir_1 | RT | CTCAACTGGTGTCGTGGAGTCCGGCAATTCAGTTGAGTCCTCCCT | |
| novel_ mir_2 | RT | CTCAACTGGTGTCGTGGAGTCCGGCAATTCAGTTGAGAAAATGGA | |
| novel_mir_4 | RT | CTCAACTGGTGTCGTGGAGTCCGGCAATTCAGTTGAGTCTGCTTG | |
| novel_mi_17 | RT | CTCAACTGGTGTCGTGGAGTCCGGCAATTCAGTTGAGCAAAATCT | |
| novel_miR_21 | RT | CTCAACTGGTGTCGTGGAGTCCGGCAATTCAGTTGAGGCCAGTTT | |
| novel_mir_53 | RT | CTCAACTGGTGTCGTGGAGTCCGGCAATTCAGTTGAGAGCCGCCG | |
| hsa-let-7i-5p | F | ACACTCCAGCTGGGTGAGGTAGTA | |
| hsa-mir-146a-5p | F | ACACTCCAGCTGGGTGAGAACTGA | |
| hsa-mir-328-3p | F | ACACTCCAGCTGGGCTGGCCCTCT | |
| hsa-mir-335-3p | F | ACACTCCAGCTGGGTTTTTCATTA | |
| hsa-mir-432-5p | F | ACACTCCAGCTGGGTCTTGGAGTA | |
| hsa-mir-663a-5p | F | ACACTCCAGCTGGGCGGGGCGCCG | |
| novel_mir_1 | F | ACACTCCAGCTGGGAGAGCTTATG | |
| novel_mir_2 | F | ACACTCCAGCTGGGGCTTGTTGTG | |
| novel_mir_4 | F | ACACTCCAGCTGGGAAAATGAAGG | |
| novel_mir_17 | F | ACACTCCAGCTGGGTTTGCTTGTG | |
| novel_mir_21 | F | ACACTCCAGCTGGGTCTACAACTG | |
| novel_mir_53 | F | ACACTCCAGCTGGGCTTCTCGAGG | |
| miR | Rev | AACTGGTGTCGTGGAG | |
| GAPDH | F | CTCATTTCCTGGTATGACAAC | |
| GAPDH | R | CTTCCTCTTGTGCTCTTGCT | |

**Supplementary data 2.** Statistical analysis of high-throughput sequencing reads for the three libraries.

|  | Control |  | Test 1 | | Test2 | |
| --- | --- | --- | --- | --- | --- | --- |
| Type | **Count** | **Percent (%)** | **Count** | **Percent (%)** | **Count** | **Percent (%)** |
| total_reads | 11400602 |  | 12235949 |  | 12366199 |  |
| high_quality | 11138629 | 100 | 12019568 | 100 | 11910647 | 100 |
| 3'adapter_null | 276454 | 2.48 | 144971 | 1.21 | 238208 | 2.00 |
| insert_null | 36112 | 0.32 | 15210 | 0.13 | 12799 | 0.11 |
| 5'adapter_contaminants | 5238 | 0.05 | 5356 | 0.04 | 5205 | 0.04 |
| smaller_than_18nt | 83030 | 0.75 | 264380 | 2.20 | 265619 | 2.23 |
| polyA | 140 | 0.00 | 876 | 0.01 | 1231 | 0.01 |
| clean_reads | 10737655 | 96.40 | 11588775 | 96.42 | 11387585 | 95.61 |

**Supplementary data 3.** Distribution of small RNAs among different categories.

|  | **Control** | | **Test 1** | | **Test2** | |
| --- | --- | --- | --- | --- | --- | --- |
| **Category** | **Unique sRNAs** | **Total sRNAs** | **Unique sRNAs** | **Total sRNAs** | **Unique sRNAs** | **Total sRNAs** |
| exon_antisense | 338 | 447 | 751 | 1571 | 1053 | 1849 |
| exon_sense | 55744 | 81781 | 48846 | 68869 | 67734 | 110823 |
| intron_antisense | 1578 | 2996 | 4756 | 16200 | 5389 | 14196 |
| intron_sense | 9403 | 13475 | 17526 | 37747 | 24307 | 38894 |
| miRNA | 3685 | 2141761 | 3651 | 1900656 | 3394 | 975144 |
| rRNA | 191043 | 3958612 | 140948 | 1966523 | 193351 | 4015757 |
| snRNA | 4986 | 35201 | 5395 | 42612 | 6183 | 48435 |
| snoRNA | 6995 | 30560 | 6319 | 24186 | 9022 | 42183 |
| tRNA | 46433 | 1857060 | 44154 | 1434700 | 35630 | 869903 |
| unann | 436298 | 2541485 | 1034446 | 5998357 | 1229690 | 5130252 |
| Total | 756503 | 10663378 | 1306792 | 11491421 | 1575753 | 11247436 |


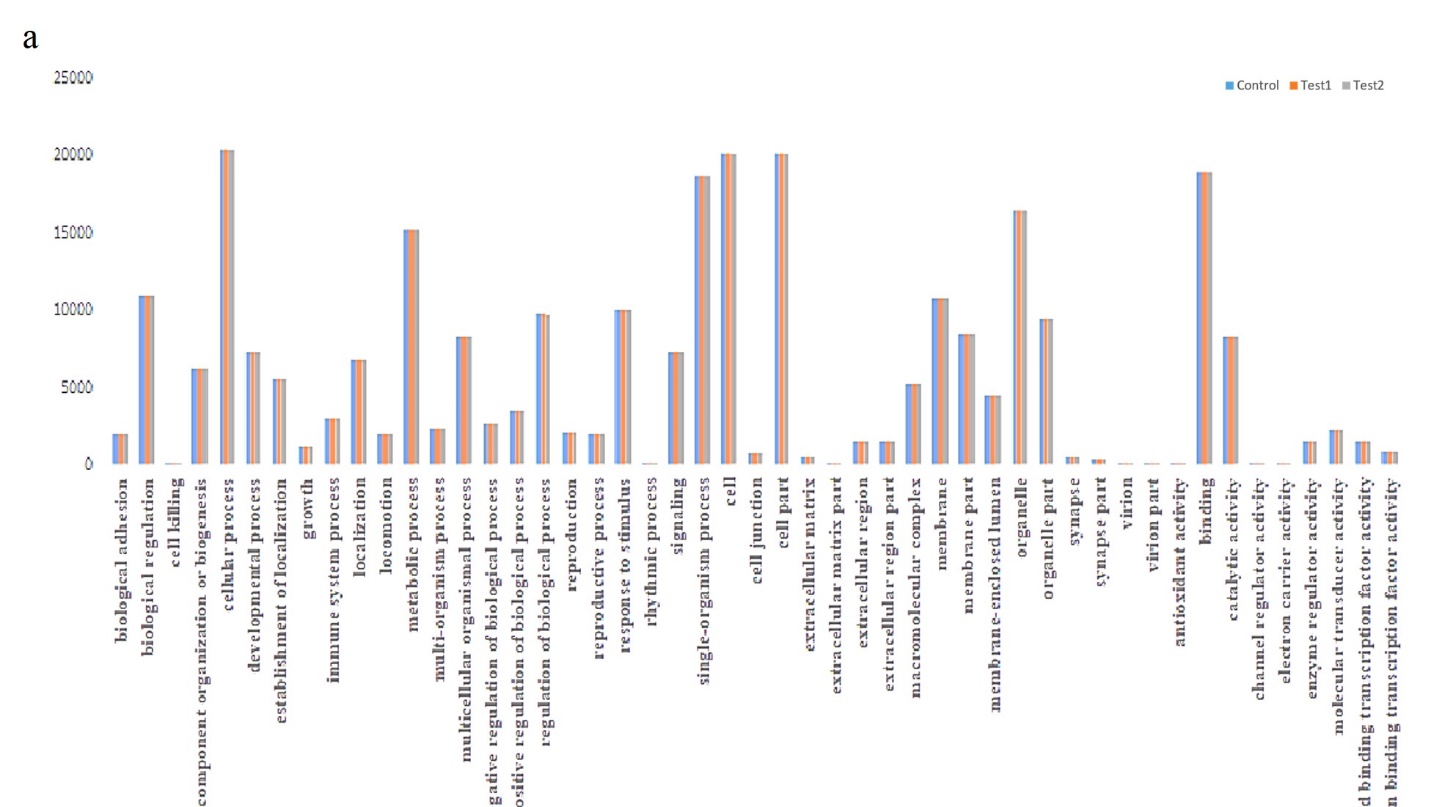


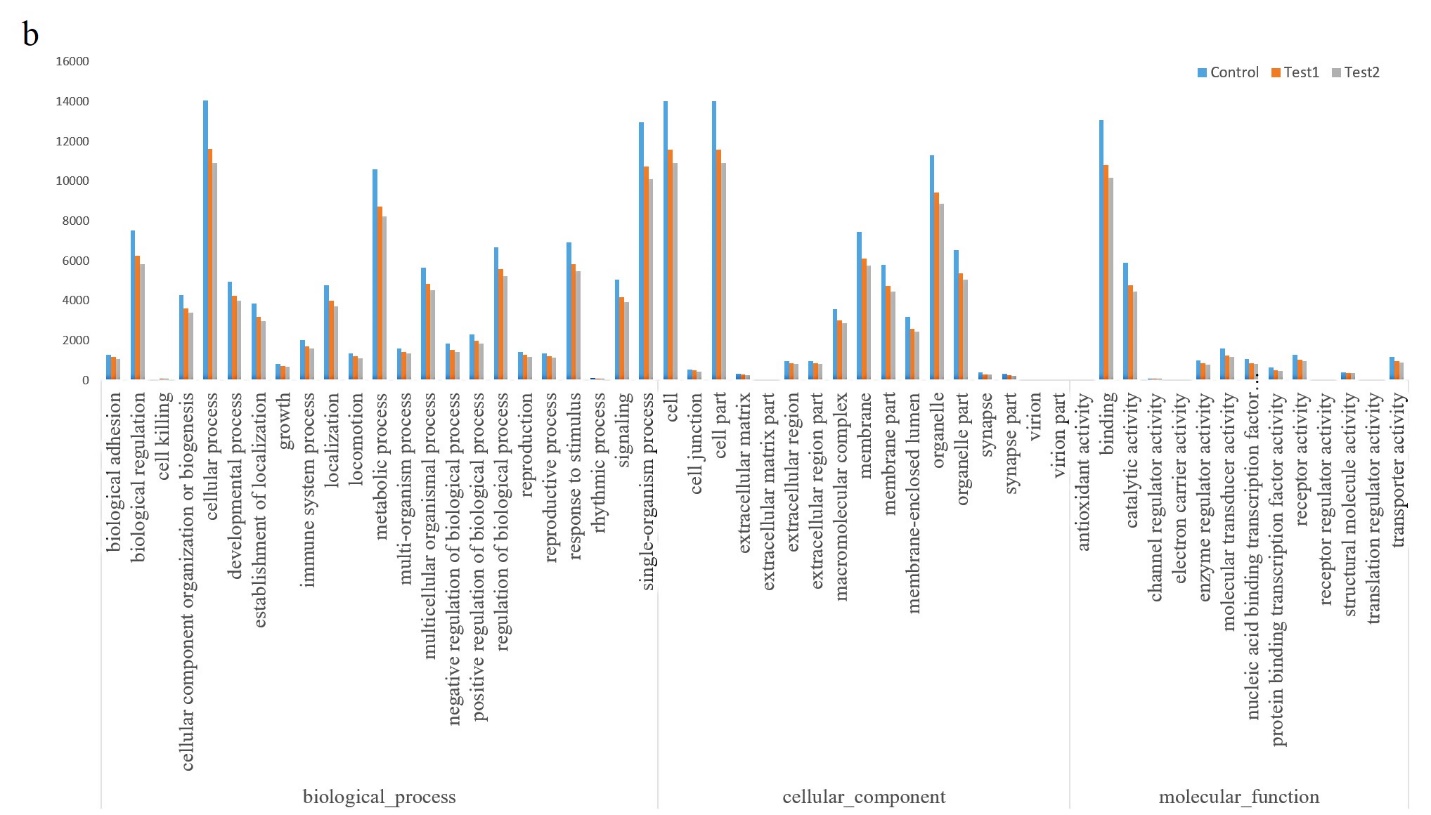


**Supplementary data 4.** miRNA target genes prediction and annotation. Target genes of conserved (a) and novel (b) miRNAs were categorized in 23, 17 and 14 classes as cellular component, biological process and molecular function, respectively. The most over-represented GO terms in the biological process were cellular process, single organism process and metabolic process. In the cellular component, however, the most over-expressed GO terms were in cell, cell part and organelle classes. Binding and catalytic activities were classes in which most of miRNAs were expressed in molecular function superclass.
